# The concept of resilience to Alzheimer’s Disease: current definitions and cellular and molecular mechanisms

**DOI:** 10.1186/s13024-024-00719-7

**Published:** 2024-04-08

**Authors:** Luuk E. de Vries, Inge Huitinga, Helmut W. Kessels, Dick F. Swaab, Joost Verhaagen

**Affiliations:** 1 Department of Neuroregeneration, Netherlands Institute for Neuroscience, Institute of the Royal Netherlands Academy of Arts and Sciences, 1105 BA Amsterdam, The Netherlands; 2 Department of Neuroimmunology, Netherlands Institute for Neuroscience, Institute of the Royal Netherlands Academy of Arts and Sciences, 1105 BA Amsterdam, The Netherlands; 3 Swammerdam Institute for Life Sciences, Amsterdam Neuroscience, University of Amsterdam, 1098 XH Amsterdam, the Netherlands; 4 Department of Neuropsychiatric Disorders, Netherlands Institute for Neuroscience, an Institute of the Royal Netherlands Academy of Arts and Sciences, 1105 BA Amsterdam, Netherlands; 5 Department of Molecular and Cellular Neurobiology, Center for Neurogenomics and Cognitive Research, Neuroscience Campus Amsterdam, VU University, Boelelaan 1085, 1081 HV Amsterdam, The Netherlands

**Keywords:** Alzheimer’s disease, Animal models, Cognition, Molecular biology, Neuropathology, Post-mortem human brain, Resilience

## Abstract

Some individuals are able to maintain their cognitive abilities despite the presence of significant Alzheimer’s Disease (AD) neuropathological changes. This discrepancy between cognition and pathology has been labeled as resilience and has evolved into a widely debated concept. External factors such as cognitive stimulation are associated with resilience to AD, but the exact cellular and molecular underpinnings are not completely understood. In this review, we discuss the current definitions used in the field, highlight the translational approaches used to investigate resilience to AD and summarize the underlying cellular and molecular substrates of resilience that have been derived from human and animal studies, which have received more and more attention in the last few years. From these studies the picture emerges that resilient individuals are different from AD patients in terms of specific pathological species and their cellular reaction to AD pathology, which possibly helps to maintain cognition up to a certain tipping point. Studying these rare resilient individuals can be of great importance as it could pave the way to novel therapeutic avenues for AD.

## Background

Alzheimer’s disease (AD) is a chronic neurodegenerative disorder causing memory loss and impairments in cognitive and behavioral functioning. AD is the most common form of dementia, which affects around 55 million people worldwide. The disease is characterized by depositions of β-amyloid (Aβ) into plaques, hyperphosphorylated tau (ptau) forming neurofibrillary tangles (NFTs), dystrophic neurites, synaptic loss and atrophy of neurons and brain regions. All these changes ultimately trigger the cognitive decline and behavioral symptoms of AD [1]. Dominantly inherited familial AD (fAD) has been linked to mutations in the genes amyloid precursor protein (APP) and presenilin 1 and 2 (PS1 and PS2), which play an important role in generation of Aβ aggregates. fAD accounts for less than 1% of all cases, while the far more common sporadic and late-onset AD (LOAD) has most likely a multifactorial aetiology including genetics, lifestyle, level of education and unknown external factors [2–4]. In the absence of effective treatments, interventions focusing on delaying the onset of AD by activation of the brain have gained more and more attention [5, 6]. Lifestyle factors like cognitive, social and physical activities might have the potential to postpone AD [7–10].

It is widely assumed that the neuropathological hallmarks of AD are causally related to cognitive decline in AD patients. This holds in particular for NFTs [11]. However, several studies have indicated a disjunction between the degree of AD pathology and its clinical manifestations. Early studies discovered that some individuals, characterized as cognitively normal, had advanced AD pathology at post-mortem examination [12, 13], which was later substantiated in cognitively intact individuals with longitudinal assessments of cognition and post-mortem assessments of pathology [14–17]. These findings led to the hypothesis that some individuals might have a “reserve” that allows them to cope with neuropathology and remain cognitively intact. Furthermore, epidemiological evidence showed a reduced risk of dementia in individuals with higher educational or occupational attainment [18, 19], IQ [20] and participation in leisure activities [21]. It was hypothesized that certain individuals exhibit progression of AD pathology while their lifelong experiences allowed them to cope better with disease-related changes.

The first aim of this review is to discuss the definitions of reserve to AD in the context of the epidemiological and experimental evidence. While it is hypothesized that the underlying mechanisms might be rooted in structural and functional brain mechanisms, more and more data on the cellular and molecular substrate of a reserve in AD have been published. Therefore, the second aim is to review the emerging cellular and molecular evidence of reserve. Insight into the cellular and molecular mechanisms that govern resilience to AD may be the starting point for the development of better treatments for AD and is therefore an important field of research. 

### Conceptual considerations

In its most essential form, a reserve allows an individual to remain cognitively intact despite the presence of extensive AD pathology. Over the years numerous terms have emerged to describe this phenomenon, such as reserve, resilience, non-demented Alzheimer neuropathology (NDAN), clinically silent AD, pre-symptomatic AD, asymptomatic AD or preclinical AD. All of these definitions have been used to describe individuals with AD pathology that are cognitively normal, but whereas preclinical AD has been primarily used in living subjects that are biomarker positive but not necessarily resilient, the former definitions have all been used for individuals at autopsy. Importantly, it has been proposed that resilience is different from resistance, which refers to the absence or lower level of AD or comorbid neuropathology relative to the expected frequency or severity based on age, genetics or other characteristics [22]. For example, as levels of Aβ plaques and ptau have been shown to increase with age, individuals at advanced ages, like centenarians, are expected to have high levels of AD pathology. However, among cognitively intact centenarians, there are individuals without any Aβ plaques in the brain, which are resistant to developing Aβ plaques. There are also cognitively intact centenarians with similar amounts of AD pathology as an demented AD patient, which are resilient [23, 24]. Researchers often attempt to describe the phenomenon of reserve from their own perspective. For example, NDAN is used by researchers using post-mortem tissue, in which cognition is not always longitudinally assessed, making it impossible to make claims about maintenance of cognition. In the field, the different terminologies mentioned here are often used interchangeably, making it difficult to align relevant mechanisms that have been found so far. To develop potential consensus definitions, a recent workgroup has subdivided reserve into three concepts: cognitive reserve (CR), brain reserve (BR) and brain maintenance (BM) [25].

The CR hypothesis postulates that individuals with a reserve process cognitive tasks in a more efficient manner. It is hypothesized that individuals with CR have increased adaptability to cope with neuropathology through specific processes, like increased functional efficiency of brain networks. BR relates to anatomical differences like a higher number of neurons, synapses or other structural brain resources by which individuals thus can withstand more atrophy or synaptic loss before clinical manifestations occur. Finally, the definition of BM has emerged to illustrate the notion of preservation of brain morphology or absence of neuropathological change over time [26]. In BM, processes like neurogenesis, repair mechanisms or removal of pathology by glial cells might play an active role.

Definitions of CR, BM and BR have been proposed as a general framework, which can be used to harmonize alternative definitions or be seen as equivalent to definitions used by other researchers. Thus far, CR, BM and BR have primarily been used in clinical studies focusing on imaging and fluid biomarkers. Lifelong experiences, such as years of education, IQ, social interactions, complexity of occupation, leisure activities and socioeconomic status, are often used as proxies for reserve as they are associated with a later onset of AD and with processes that can be linked to the proposed definitions. For example, more years of education has been linked to larger brain volumes [27, 28], related to BR. However, the use of such proxies often results in a biased measure and affects outcome measures independent of reserve. For example, more years of education has been directly linked to better health [29], and individuals with higher education levels may perform better in cognitive status questionnaires. Furthermore, the role of these proxies in resilience remains unclear as it was recently demonstrated that IQ explains more variation in rate of cognitive decline than years of education [30], suggesting that IQ might be a better proxy for resilience than years of education. To overcome these issues, more recent studies have used multiple proxies to combine lifelong exposures that are related to resilience into a composite score [31–33]. In addition, others have implemented more direct measures related to resilience and neural mechanisms by using functional magnetic resonance imaging (fMRI) or changes in fluid biomarkers to identify alterations in network activity or relevant brain changes, respectively. This approach has been labeled the “neural implementation” of CR [25].

Researchers focusing on molecular and cellular mechanisms often use the definitions resilience or resistance, which, according to the proposed framework, could be attributed to CR or BM, respectively. The notion of resilience has also been recapitulated in animal models by identifying animals with better cognition than expected based on age or pathology [34, 35]. Repetitive cognitive training or enriched environment improved cognition in animal models of AD [36, 37], underlining the idea that increased plasticity through cognitive training contributes to resilience. The influence of positive novel experiences (such as enriched environment) or negative experiences (stress, early life adversity) on cognition has been well established in rodents [38–41]. Researchers often attempt to characterize mechanisms that have been identified in animal studies as either CR or BM. For example, in young and aged rats, which were either cognitively impaired or unimpaired based on behavioral tests, genes related to age were correlated with cognition to identify genes related to CR [42]. The authors concluded that upregulated genes, counteracting aging stressors that impair cognition, such as neuroinflammation and oxidative stress, and downregulated genes, related to nervous system development, reflect adaptive changes in the circuit to preserve cognition. However, when focusing on such complex molecular or cellular mechanisms, it becomes difficult to relate the mechanism back to the proposed definitions. For example, neuroinflammation in the ageing brain can both be linked to exacerbation of neuropathology or to synapse pruning [43], and thus influences both CR and BM.

When focusing on the molecular and cellular mechanisms of resilience, an analogy can be made to what has been called the cellular phase of AD [44]. In this concept there is a complex cellular phase, in which cells respond to Aβ and ptau aggregations. In this phase, which possibly could last for decades, there is a gradual shift from the initial reversible physiological reactions to pathology to irreversible compensation mechanisms, which could be independently of Aβ and ptau. This would consequently disturb the brain homeostasis and lead to clinical symptoms. In resilience, cellular reactions to pathology might not develop into irreversible changes, resulting in maintenance of brain homeostasis. The absence of some of these irreversible cellular changes can be linked back to CR and BM, such as loss of synaptic inputs evolving into alterations in connective patters or the initial clearance of pathology evolving into clearance dysfunction. The proposed definitions are well suited to describe changes on the macroscopic level (e.g. changes in brain volume or circuit changes) or microscopic level (changes in the amount of pathology or the amount of synapses). However, the possible upstream molecular mechanisms orchestrating the observed changes in CR and BM are often complex, rooted in both types of reserve and not fully elucidated. Upstream effectors such as transcription factors might have such a broad effect that it will influence both resilience or resistance, or CR and BM, making it impossible to distinguish between the two. Hence, when focusing on CR and BM, there is a possibility that the downstream effects of the cellular and molecular mechanisms are observed, but not the fundamental mechanism itself.

When investigating molecular effects to such an extent, their link to phenotype is sometimes difficult to establish. Studies focusing on molecular effects of Aβ and tau on individual cells often refer to cellular resilience, in which specific cell types are not affected by pathology. For instance, in post-mortem tissue, different excitatory neuron subtypes were found to be more resilient to tangle formation [45], or altered levels of proteins such as mitofusin 2 (MFN2) or RAR Related Orphan Receptor B (RORB) were associated with resilience or vulnerability to tangles [46, 47]. These resilient cellular subtypes or mechanisms cannot always be linked back to phenotypic traits, such as cognition. Furthermore, the challenge with these studies is to translate a cellular view of resilience back to an overall view of resilience in an organism. It is often unknown how molecular changes in specific cells, often studied in only one brain area, influence a potential brain-wide phenomenon. Currently, molecular and cellular mechanisms are often attributed to resilience or resistance due to correlations with cognition or the amount of pathology. However, causal evidence of these mechanisms on behavioral phenotype (cognition) or biochemical phenotype (pathology) is often incomplete. Studies elucidating if these complex cellular and molecular responses can be untangled into separate mechanisms related to CR and BM are required to further understand how they contribute to cognition.

While it is evident that some individuals exhibit resilience to AD, a clear cellular or molecular substrate is lacking. We propose to use resilience as a general term when focusing on complex cellular and molecular substrates of reserve in AD, and cellular resilience when there is resilience on a cellular level, for example neuronal subtypes without tangle formation, which cannot be traced back to cognition. Only when both causal evidence and effects on phenotype are present, molecular and cellular processes can be attributed to CR or BM, or resilience and resistance, respectively. Possible mechanisms related to resilience rooted in cellular and molecular mechanisms are often derived from translational approaches in animal models or from human post-mortem studies, which will be highlighted in the next sections of this review.

### Strengths and limitations of translational approaches

Most evidence for resilience to AD has been obtained in large-scale longitudinal cohorts by investigating if there is a better cognitive performance as could be expected based on the amount of pathology, inferred from the analyses of blood or cerebrospinal fluid (CSF), of brain imaging or of post-mortem neuropathology. More recently, animal models have been used to discover potential mechanisms of resilience. While each of these approaches have their advantages and disadvantages, all are required to help understand the molecular basis of resilience.

**Fig. 1:**
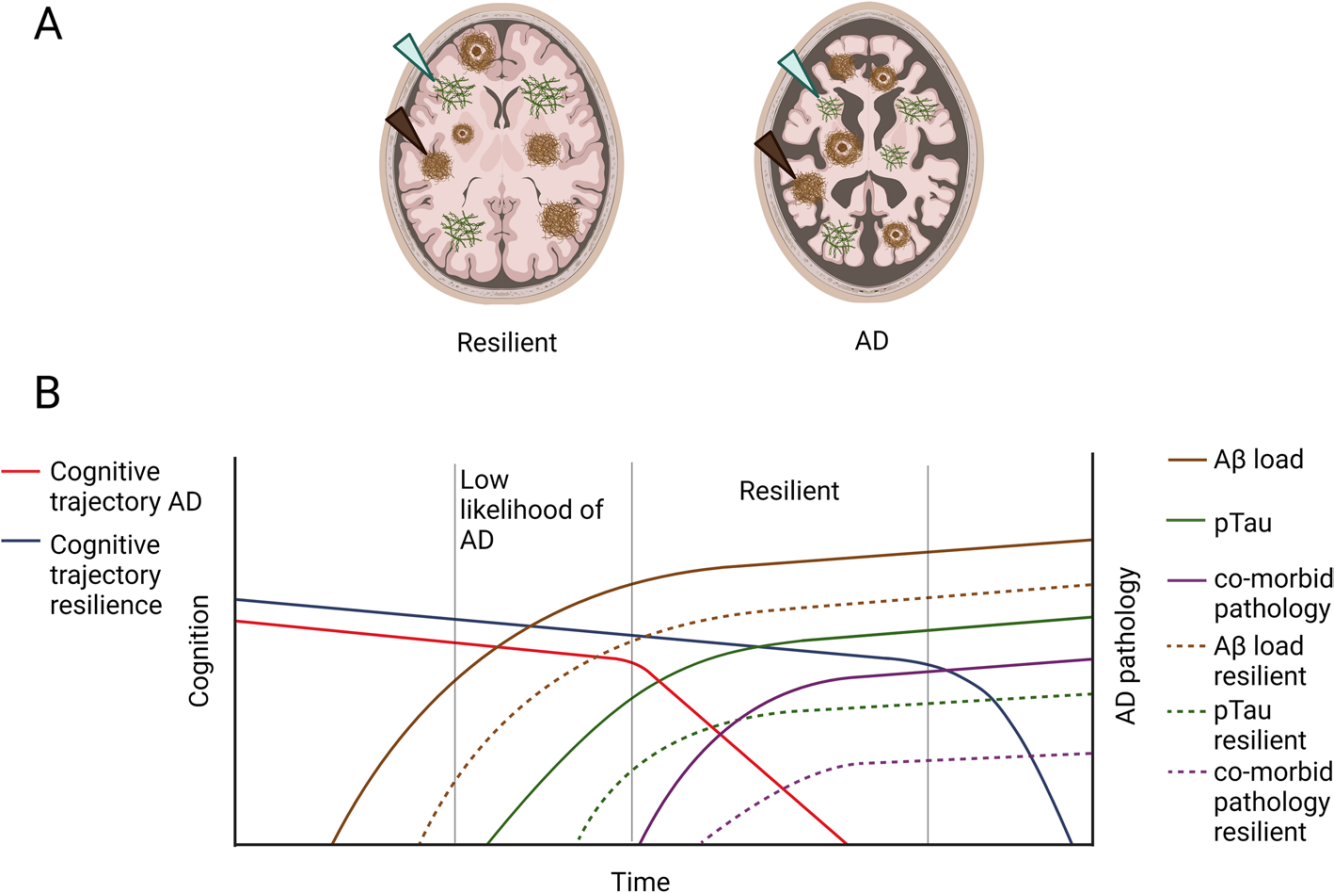
Representation of cognitive aging in the presence of AD neuropathology in AD-patients and resilient individuals. Simplified overview of the AD neuropathological burden versus cognitive functioning during aging or disease progression. **A** Schematic of a resilient and AD brain with the neuropathological hallmarks of AD, neurofibrillary tangles (green arrows) and amyloid-beta plaques (brown arrows) and atrophic brain regions, as can be seen by the shrinkage of gyri and sulci and larger volume of the vessels, in AD. **B** Schematic of the progression of cognition and AD pathology over time. Plaques are present years before the onset of the disease and increase over time (brown line). The onset of phosphorylated tau (green line) follows and corresponds better with clinical progression in AD (red line). In later stages of the disease, comorbid pathology is often found in AD, such as TAR DNA-binding protein 43 (TPD-43) inclusions, α-synuclein (α-syn) or hippocampal sclerosis (purple line). It is hypothesized that resilient donors have a similar progression of AD pathology but are able to stay cognitively intact for a longer period (blue line). Multiple studies have also shown that in fact resilient donors are also to a certain extend resistant, as they have reduced amounts of AD pathology and comorbid pathology (dashed lines). Importantly, when low to intermediate amounts of AD pathology are present in the brain, it is difficult to differentiate resilient donors and those who would progress to dementia. Thus, in that timeframe it is difficult to assign resilient donors, which is here depicted as low likelihood of AD based on the amount of pathology

The most accurate method to diagnose AD pathology and comorbidities is by post-mortem neuropathological analysis. It is essential to perform a neuropathologic diagnosis on post-mortem tissue, as common co-morbid neuropathological changes, for instance related to hippocampal sclerosis or early Parkinson’s disease, also influence cognition. To illustrate this point, Montine et al. [48] have defined *apparent resilience*, referring to specific lesion types measured in vivo with positron emission tomography (PET) scans without consideration of common co-morbidities, and *essential resilience,* which can only be determined through post-mortem neuropathologic assessment. An individual with apparent resilience might be cognitively intact due to the absence of common comorbid pathology, while a demented individual with similar amounts of AD pathology might have these comorbidities. The authors demonstrated that most cases of apparent resilience were in fact also classified as resistant to co-morbid disease, i.e. resilient as they remain cognitively intact despite presence of AD pathology and resistant to pathological comorbidities as these were lower than expected based on age and levels of AD pathology. Whereas clinical and pathological data are both crucial in determining whether a donor is resilient to AD pathology, often these data are incomplete or not adequately described in post-mortem studies. Unfortunately, most longitudinal cohort studies that test cognitive capabilities over time often have long post-mortem delays, while other studies or brain banks have more specialized neuropathological protocols [49], but have limited clinical information or lack longitudinal measurements of cognition. Another important caveat is that different pathological AD staging systems are used resulting in different subpopulations that are being studied, which is often the result of the scarcity of true resilient donors. Whereas some studies strictly classified resilient individuals with only high amounts of AD pathology [Braak VI [50] and CERAD ≥ 2 [51], most researchers have pooled cognitive intact individuals with Braak stages III-VI [52]. Importantly, different glial subtypes were recently demonstrated in the visual cortex in post-mortem tissue of resilient versus AD patients at Braak stages III-IV [53]. This study demonstrates that cellular and molecular changes can happen before the appearance of tangle formation in the occipital cortex. It remains however uncertain if donors with lower amounts of AD pathology would remain cognitively intact if pathology would progress further. Some donors might remain cognitively intact while others would progress to dementia, which might confound the comparison of AD-related effects between resilient and AD-patients (Fig. 1). While the use of post-mortem tissue allows the implementation of omics on a cellular and molecular level, several confounders that generally lead to unwanted variability must be taken into consideration, including post-mortem delay, agonal state, fixation methods or medication. In addition, due to the absence of a clear biomarker for resilience, the relation between measures for cognition and for pathology are used to identify resilience. As it has been estimated that up to 40% of variance in cognition is not explained by AD pathology or risk factors [54], other parameters than pathology and cognition likely play a role. Once a clear biological substrate for resilience has been established, these other factors, which are likely independent of Aβ and ptau, should become more apparent. Finally, it is important to realize that post-mortem tissue provides a snapshot of the molecular mechanisms and pathology at time of death, which might deviate from a longitudinal assessment. With the development of novel PET and fluid biomarkers for specific features such as Aβ and ptau burden, neuronal damage, α-synuclein, or glial markers, resilience and resistance can be estimated more accurately and longitudinally in living subjects and possibly validate molecular and cellular changes initially discovered in post-mortem tissue. As investigating if resilience mechanisms are causal or simply correlate with resilience is currently not possible in human subjects, others have tried to study the concept of resilience in animal models.

To date, it remains difficult to model the concept of resilience in animal models. One model that tries to recapitulate the idea of the positive effects of lifestyle factors on cognition is enriched environment (EE). Several studies have shown beneficial effects after EE, including improved cognition and a reduction of the amount of both Aβ and ptau [55–57]. Similar to the human situation, it remains unclear which lifestyle factors or factors associated with EE, such as social interaction, cognitive stimulation, novelty or exercise, contribute to these beneficial effects. Importantly, while exercise in rodents has shown robust effects on cognition, EE without exercise has also shown to protect from age-related cognitive decline [58]. Some researchers have used a different approach by identifying learners and non-learners in aged animal populations or in AD animal models. These studies found a role for neurogenesis by showing activity dependent activation of newborn neurons in aged animals and the activation of phospholipase A2 (*PLA2G4E*) in the Tg2576 model by comparing learners to non-learners [35]. Another strategy that has recently been put forward is trying to reflect the genetic diversity of humans in the 5xFAD model by crossing it with BXD mice, which are a series of recombinant inbred strains [59]. By using such an approach, different transcriptional networks enriched in astrocyte and microglia markers have been identified as drivers of resilience in AD [60]. Interestingly, these networks showed a considerable overlap with previously identified networks in the human brain [61], underlining that genetics play an important role in resilience. In addition, animal models provide an excellent opportunity to investigate cellular resilience. Mechanisms that allow cell types or synaptic components to become resilient to Aβ or ptau can be identified in well-controlled environments. For instance, in the APP/PS1 model, it was shown that Aβ-induced deficits in learning and memory and synaptic plasticity depend on α-amino-3-hydroxy-5-methyl-4-isoxazolepropionic acid (AMPA) receptor subunit GluA3, rendering synapses without GluA3 resilient to Aβ [62].

Ultimately, an interdisciplinary approach is required to elucidate how educational attainment and lifestyle factors can lead to structural and functional changes on both a macroscopic, cellular and molecular level. More large-scale longitudinal community cohorts are required to adequately identify resilient donors with longitudinal cognitive testing and autopsy with a short post-mortem delay and extensive neuropathological characterization. One example is the longitudinal centenarian cohort [63]. Importantly, findings derived from post-mortem studies should be validated in vitro or in vivo to demonstrate causality and link them to either CR or BM, or resilience or resistance, respectively. To recognize the different strengths and limitations discussed above, the most important papers discussed in this review are summarized in Table 1, indicating how resilience was determined in each study.

**Table 1:** Pivotal studies of molecular and cellular studies on resilience in AD

Study	Main focus	Brain regions	Resilient cases (*N*)	Key findings	Amount of AD pathology	Remarks on the inclusion determinants of resilience
Arenaza-Urquijo et al., 2019 [64]	FDG-PET in resilient donors	FDG-PET	Large cohort n = 192	FDG-PET uptake in bilateral anterior cingulate cortex and anterior temporal pole was associated with baseline cognition despite presence of established AD biomarkers	-	Resilience based on cognition over time, ranging from 2 to 10 years and PiB-PET signal
Azarpazhooz et al., 2019 [52]	Systemic review on prevalence of resilient donors in community dwelling elderly	Diagnostic neuropathological assessments	Pooled from 17 large cohorts	Significant portion of population is resilient to a certain extend	CERAD none-frequent, Braak 0-VI	Large sample size but wide spread of pathology (low and high amounts of AD pathology mixed in the resilient group)
Arnold et al., 2013 [65]	Cellular and synaptic proteome	Medial prefrontal cortex	10	Preservation of synaptic density and neurons, decrease in astrocytes. In addition, alterations in apoptosis, cell-cycle proteins and ion transport	CERAD moderate – frequent, Braak III-V	Excluded cases with pathological comorbidities (abundant LBs, infarcts, HS), reduced amount of NPs in resilient versus AD, annual cognitive testing
Barker et al., 2021 [66]	MEF2C	Anterior prefrontal cortex	9	Upregulation of MEF2C and an enrichment of MEF2C target in cortical excitatory neurons of resilient individuals. MEF2 increased after EE and overexpression rescues cognitive deficits in tauopathy mouse model	CERAD none – frequent, Braak III-V	Included only female cases, PMD ≤ 20 h, four resilient cases exhibited no amyloid pathology, annual cognitive tests
Barroeta-Espar et al., 2019 [67]	Cytokine and growth factors	Entorhinal cortex and superior temporal sulcus	11	Protein analysis of the cytokine profile in resilience was associated with enhanced neuroprotection, reduced activation of glial cells (GFAP and CD68)	CERAD, moderate – frequent, Braak V-VI	Both high probability and low probability resilient donors used, which both differ from AD, cognition assessed within 2 years prior to death or by absence of MCI or dementia in clinical records
Bilousova et al., 2016 [68]	Synaptic oligomeric burden	Parietal cortex, superior parietal cortex, entorhinal cortex and hippocampus	16	p-Tau and Aβ oligomeric burden in synaptosomes was only present in later stages of AD pathology and not in the non-demented (resilient) cases	CERAD non-moderate, Braak III-IV	Proportion of resilient donors with cognitive impairments, resilient donors compared to both early and late AD (based on Braak stage), cognition based on MMSE
Bjorklund et al., 2012 [69]	Postsynaptic localization of Aβ oligomers	Dentate gyrus and CA3	10	LMW Aβ oligomers were shown to be elevated at the post-synaptic density of demented patients. Reduced ZnT3 mRNA, resulting in an increase in Zn^2+^ in the synaptic cleft, facilitating LMW Aβ oligomers to bind	CERAD moderate, Braak V (only median)	Moderate density of neuritic plaques (CERAD = B), cognition based on MMSE
Boros et al., 2017 [70]	Dendritic spines	Dorsolateral prefrontal cortex	8	Resilient individuals showed an overall increase in dendritic spine density and extent	CERAD Moderate – frequent, Braak I-IV	No or sparse frontal NFTs compared to dementia cases with moderate/frequent NFTs Cognition based on MMSE
Briley et al., 2016 [71]	Neurogenesis	Dentate gyrus	4	Number of SOX2 cells increased in resilience, increased expression of miRNA’s related to neurogenesis	CERAD NA, Braak IV-V	PMD of 40 h in one case, majority of post-mortem tissue from females, n is low, cognition based on MMSE
Buciuc et al., 2020 [72]	Comorbid AD pathology	Diagnostic neuropathological assessments	21	Lower frequency of TPD-43 pathology in resilient compared to non-resilient donors	CERAD Moderate – frequent, Braak IV-VI	AD and resilient cases matched well based on CERAD and Braak, annual cognitive testing
Cain et al., 2023 [73]	Cellular states and communities related to AD and cognition	DLPFC	6	Model cellular states and communities derived from snRNA-seq. donors in a larger dataset of bulk-RNAseq. Specific cellular communities are related to cognitive decline with high amounts of ptau or maintenance of cognition with lower levels of ptau	CERAD and Braak NA (AD diagnosis confirmed by neuropathologist) Quantified AD pathology in eight different brain regions	Annual cognitive tests, higher ptau load in AD samples compared to resilient samples
Carlyle et al., 2021 [74]	Proteomics of synaptomes	Angular gyrus	25	Proteins involved in the serotonin neurotransmitter release and oxidative phosphorylation were upregulated in resilience. Glycolysis, glutathione metabolism and proteasome components were downregulated	CERAD NA, Braak V	Three cases with Lewy Body pathology, resilient individuals exhibit a significantly lower global AD pathology compared to demented patients, annual cognitive tests
Dumitrescu et al., 2020 [75]	GWAS for resilience	Amyloid PET screening or autopsy based diagnostics	337 autopsy 2980 PET	Single SNP related to resilience upstream of ATP8B1	CERAD none-frequent, Braak NA	Resilience based on amyloid-plaques (neuritic plaques) or PET signal, annual cognitive testing
Eissman et al., 2022 [76]	Sex-specific GWAS for resilience	Amyloid PET screening or autopsy based diagnostics	5024 PET and autopsy	Female-specific SNP in chromatin loops that interact with genes related to RNA-processing (GATA3)	CERAD none-frequent, Braak NA	Extensive cognitive tests, harmonized cognitive data across the different cohorts, resilience based on NPs or PET signal
Esparza et al., 2013 [77]	Aβ oligomers	Frontal and parietal cortex	24	Reduced amounts of oAβ in resilient compared to mild AD	CERAD and Braak NA, Resilient and AD donors stratified by % plaques (IHC)	Cognition determined based on CDR (0 for resilient and 1 for AD)
Fracassi et al., 2023 [78]	Microglia activation	Frontal cortex	6	Hyperactive microglia around resilient plaques, based on TYROBP, TREM2 and CD68	Braak III-VI	Braak lower in resilient than AD, unclear which resilient donors were used, cognition based on MMSE
Heuer et al., 2020 [60]	Co-expression modules in AD-BDX animals related to cognition	Hippocampus	71	Fgf2 potential regulator of networks related to working memory and short term memory	5xFAD mice crossed with AD-BDX panel	Cognition based on y-maze, and fear conditioning paradigms at 6 or 14 months of age
Hurst et al., 2023 [79]	Proteomic networks on cortical resilient tissue	Frontal and temporal cortex	53	NR1N identified as regulator of protein network related to synapses based on proteomic data, NR1N prevents AB-induced spine loss and synaptic plasticity hyperexcitability	CERAD moderate-frequent, Braak II-VI	Increased MAPT/tau burden in AD compared to resilient, cognition based on MMSE
Iacono et al., 2008 [80]	Nuclear hypertrophy	Anterior cingulate gyrus, posterior cingulate gyrus, primary visual cortex	15	Hypertrophy of neuronal cells in CA1 of hippocampus and anterior cingulate gyrus in resilient versus AD and control donors	CERAD moderate-frequent, Braak 0-IV	Resilient donors fewer neuritic and lower Braak stages than demented groups, cognition based on multiple cognitive tests
Jiang et al., 2021 [81]	Synaptotoxicity by Aβ- and tau	Frontal cortex	6	No significant differences in soluble high molecular weight Aβ oligomers and soluble ptau	NA	Unclear what pathological load is between groups, cognition based on CDR
Johnson et al., 2020 [82]	Proteomics	Frontal cortex and temporal cortex	53	Dementia associated with upregulation of MAPK/metabolism, ubiquitination, axonogenesis and downregulation of postsynaptic density proteins, mitochondria, and RNA splicing	CERAD, moderate – frequent, Braak III-VI	Cognition based on MMSE or CDR (MMSE≤24 or CDR≥1 for AD).
Kobayashi et al., 2018 [83]	Reactive astrocytes	Entorhinal cortex	10	GLT-1 reactive astrocytes are associated with a preserved cognition in the presence of AD pathology	CERAD non-frequent, Braak III-V	Cognition based on CDR, (control and resilient = 0 – 0,5, AD = 2–3). Pathology well matched between AD and resilient cases
Latimer et al., 2019 [84]	ptau burden and LATE-NC	Many cerebral regions and dentate gyrus	7	Resilient donors exhibited a lower cortical ptau burden, plaque burden and less LATE-NC	CERAD, frequent, Braak IV	Resilience is matched to demented patients with a similar ABC score, but demented patients show a significant increase in LATE-NC and macroinfarcts, cognition tested every two years with CASI
Lee et al., 2019 [85]	Network activity	116 different regions		Network efficiency related to resilience and the right middle-temporal pole might center for neural substrates	Based on Tau and Aβ PET	Network efficiency related to resilience determined by residual approach of resilience (estimating difference between actual and estimated performance of cognition based on pathology, atrophy)
Lee et al., 2021 [86]	ptau burden by deep learning technique	Medial frontal cortex and amygdala	7	Resilient donors exhibited a lower cortical ptau burden (despite ptau in neurites) and reduced LATE-NC	CERAD frequent, Braak VI	CERAD and Braak matched between AD and resilient donors, cognition based on CASI within 2 years of death
Maarouf et al., 2011 [87]	Biochemical assessment	Frontal cortex	8	Resilient individuals lack Aβ-related biochemical changes and the authors suggested that not only Aβ but additionally ptau and microvascular dysfunction play a role in cognitive deterioration	CERAD, moderate – frequent, Braak III-V	Did not included healthy controls as reference MMSE ≥ 27 for resilient donors and ≤ 19 for the AD group while the Braak and WMR was slightly lower in the resilient group
Mathys et al., 2023 [88]	Single nucleus RNA-sequencing	Frontal cortex	Large community sample n = 427	Resilient individuals have a higher proportion of inhibitory subtypes	CERAD moderate – frequent, Braak II-VI	Used cognitive resilience score (CR score) as the difference between observed cognition and cognition predicted by a linear regression model, based on global AD pathology and separately for neuritic plaque burden, neurofibrillary tangle burden and tangle density
Neuner et al., 2017 [89]	Proteomics in resilient 5xFAD mice	Hippocampus	4	Changes in neuronal excitability and synaptic plasticity, HDAC4 and REST	High plaque load expected at 8 months of age, pathology has not been investigated	Resilient 5xFAD animals based on contextual fear memory at 8 months of age, N is low
Montine et al., 2022 [90]	Comorbid pathology	Diagnostic neuropathological assessments	Large community sample n = 367	Mainly LATE-NC influences cognition	Full neuropathological assessment of 90 + study	Clinicopathologic correlations with donors which had cognitive testing in last 6 months (n = 260)
Perez-Nievas et al., 2013 [91]	Resilience phenotype	Superior temporal sulcus	8	Resilient individuals show a preservation of neurons, synapses, and axons. In addition, they have a lower plaque and oligomeric Aβ burden and not soluble ptau accumulation was observed in the synapses	CERAD frequent, Braak V-VI	Divided resilient donors in intermediate probability and high probability, high probability same pathological load as AD cases, annual clinical testing or MMSE within 2 years of death
Perez-Gonzalez et al., 2020	Transcriptomics on cognitively intact Tg2576 animals	Hippocampus	4–5	PLA2G4E is upregulated in resilient Tg2576 animals, overexpression of PLA2G4E increased dendritic spines and improved cognition in APP/PS1 animals	No differences in amount of pathology in resilient and non-resilient animals	Cognitively intact Tg2576 animals at 16–18 months of age identified with Morris water maze, N is low
Ramos-Miguel et al., 2021 [92]	Proteomics on large community cohort	Prefrontal cortex	Large community sample n = 1209	Alterations in SNARE proteins associated with resilience	Ranging from none to high amounts	Proteins related to resilience identified by controlling for cognition and pathology in large-community sample, annual cognitive tests
Scheff et al., 2016 [93]	Synaptic protein levels and oxidative stress	Hippocampus	15	No change in pre- and post-synaptic proteins and trend to lower levels of markers related to oxidative stress between resilient and MCI donors	CERAD moderate-frequent, Braak III-V	Cognition based on MMSE score, divided resilient donors in low pathology and high pathology groups, compared with individuals with MCI, amount of pathology not quantified
Seyfried et al., 2017 [94]	Network analyses of transcriptomics and proteomics	Dorsolateral prefrontal cortex and precuneus	15	Resilience was associated with an upregulation of ECM proteins, and downregulation of the synaptogenesis and synaptic function	CERAD sparse -frequent, Braak II-VI	Cognition based on annual testing, resilient donors can be characterized intermediate to high amounts of AD pathology
Silva et al., 2014 [95]	DNA damage, cell-cycle, and cell death	CA1	12	Immunohistochemistry of TMA reported more DNA repair markers and cell cycle inhibitors in resilience, but also a reduction in DNA damage and apoptosis	CERAD moderate – frequent, Braak ≥ III	Cognition based on CDR score, average lower Braak in resilient compared to AD cases
Singh et al., 2020 [96]	Synaptic integrity and ptau oligomers	Frontal cortex and hippocampus	13	Decreased oligomeric ptau in the synaptosomes of resilient individuals correlated with a preserved synaptic integrity, in addition the cortical and hippocampal synaptosomes of resilient individuals indicated preservation or increase in GluA1	CERAD: NA (N = 6), moderate (N = 2), frequent (N = 5), Braak IV-VI	Cognition based on MMSE score, plaque status was NA for several cases, Braak stages on average lower in resilient compared to AD cases
Taddei et al., 2022 [53]	IHC of glial phenotypes and cortical cell damage	Temporal and occipital cortex	20	Demented patients show an elevation of the inflammatory-induced glial-phenotype together with an increase of cellular damage in brain areas with and without NFT distribution	CERAD none ‑ frequent, Braak III-IV	Cognition based on MMSE score, CERAD and Braak score of resilient similar to AD cases
Taddei et al., 2023 [97]	Tau oligomers in synaptosomes	Occipital cortex	13	Demented patients showed synapse losses, higher proportions of internalized synapses in glial cells and more synapses with oligomeric tau compared to both resilient and control donors. In demented donors, proportion of tau containing synapses in glial cells was higher compared to control donors	CERAD none ‑ frequent, Braak III-IV	Cognition based on MMSE score, CERAD and Braak score of resilient similar to AD cases
Walker et al., 2022 [98]	Proteomics of NFT-bearing neurons and their micro-environment	Posterior hippocampal CA1	8	GeoMx™ DSP, containing 86 antibodies revealed 11 proteins that were differentially expressed in NFT-bearing neurons of the resilience, indicating a decrease in energetic and oxidative stress and a maintained synaptic integrity by intact axons, dendrites, and synapses	CERAD sparse – frequent, Braak IV-VI	Cognition based on MMSE (≤19 for AD, ⪰26 for resilient, two resilient outliers with MCI, MMSE ⪰19), CERAD higher in AD
Yu et al., 2020 [99]	Proteomics	Dorsolateral prefrontal cortex	Large community sample n = 391	Eight cortical proteins identified with resilience: NR1N, EPHX4, RPH3A, SGTB, CPLX1, SH3GL1 and UBA1	Ranging from none to high amounts	Proteins related to resilience identified by correlating with cognition based on annual cognitive tests and AD (comorbid) pathology in a large sample size
Zolochevska et al., 2018 [100]	Proteomics of the hippocampal PSD	Medial part of the hippocampus CA1	8	MALDI MS/MS, 2DE and Western blot revealed that most protein alterations were related to the cytoskeleton, calcium signaling or PTM	CERAD NA, Braak ≥ IV	Cognition based on MMSE, although high spread of MMSE scores in AD group (12–28), similar braak between resilient and AD cases
Zammit et al., 2022 [101]	Proteomics	Dorsolateral prefrontal cortex	Large community sample N > 1000	Identified 52 proteins related to resilience, involved in mitochondria, synaptic signaling and related to cell structure and function	Ranging from none to high amounts	Proteins related to resilience identified by correlating with cognition based on annual cognitive tests and AD (comorbid) pathology in a large sample size

*Abbreviations: ABC; ATP8B1* ATPase phospholipid transporting 8B1, *CASI* cognitive abilities screening instrument, *BA* brodmann area, *CDR* clinical dementia rating, *CD68* cluster of differentiation 68, *CERAD* consortium to establish a registry for Alzheimer’s Disease, *CPLX1* complexin 1, *DLFPC* dorsolateral prefrontal cortex, *EE* enriched environment, *ECM* extracellular matrix, *EPHX4* epoxide hydrolase 4, *FDG-PET* fluorodeoxyglucose-positron emission tomography, *Fgf2* Fibroblast growth factor 2, *GFAP* glial fibrillary acidic protein, *GLT-1* glutamate transporter-1, *GWAS* genome-wide association study, *HS* hippocampal sclerosis, *LATE-NC* limbic-predominant age-related TDP-43 encephalopathy neuropathologic change, *LBs* Lewy bodies, LMW: low molecular weight, *MCI* mild cognitive impairment, *MEF2*, *miRNA* microRNA, *MMSE* mini-mental state examination, *MS* mass-spectrometry, *NA* not available, *NPs* neuritic plaques, *NFT* neurofibrillary tangles, *NR1N* neuritin 1, *TMA* tissue microarray, *TMT* tandem mass tag, *pCREB* phosphorylated CREB, *PMD* post-mortem delay, *PTM* post-translational modifications, *RPH3A* rabphilin 3A, *ptau* hyperphosphorylated tau, *SGTB* small glutamine rich tetratricopeptide repeat co-chaperone beta, *SH3GL1* SH3 domain containing GRB2 like 1, endophilin A2, *SOX2* SRY-Box transcription factor 2, *snRNA* single nucleus RNA sequencing, *TREM2* triggering receptor expressed on myeloid cells 2, *TYROBP* tyrosine kinase binding protein, *UBA1* ubiquitin like modifier activating enzyme 1, *WMR* white matter rarefactions, *ZnT3* zinc transporter 3

### AD pathology in reserve

#### Identification of resilience

Evidence for the existence of resilience to AD has mainly come from large community-based cohorts with longitudinal measures of cognition and neuropathological examinations at autopsy. These large cohorts like the Religious Orders Study (ROS), the Rush Memory and Aging Project (MAP), the Nun study or the Baltimore Longitudinal Study of Aging (BLSA) have all shown a discordance between the degree of post-mortem AD pathology and ante-mortem cognition [102–104]. Similar patterns have been observed in other large cohorts, like the 90 + study [105], the Honolulu-Asia Aging Study (HAAS) [106], and the Medical Research Council Cognitive Function and Ageing Study (CFAS) [107]. In addition, it has been estimated that around one-third of the community-dwelling elderly with intermediate and high levels of AD neuropathology remained cognitively unimpaired [52]. Thus, a considerable number of the elderly population shows a discrepancy between their cognition and AD pathology. Recently, case-reports have emerged of extreme resistant or resilient cases, in which individuals with fAD were able to maintain cognition up to three decades after the expected onset of symptoms. While these individuals were resilient towards Aβ, there was either regional resistance towards tau pathology [108], demonstrated by low levels of ptau in the frontal cortex and hippocampus [109], or resilience to ptau, demonstrated by the more extensive load throughout the brain [110]. Even though a final neuropathological diagnosis of AD is required in addition to the clinical symptoms, developments in the identification of CSF and neuroimaging biomarkers [111] have allowed monitoring the progression of AD in vivo. In line with reports that extensive Aβ deposits are found in the brain at autopsy in cognitively intact individuals, multiple studies have shown that 20–40% of cognitively intact individuals over the age of 65 have Aβ biomarkers (measured by PET Pittsburgh Compound-B (PiB) uptake) above the diagnostic threshold in at least one AD vulnerable brain region [112–114]. This has also been extended to different CSF biomarkers, where AD signatures of these markers were above diagnostic thresholds for cognitively intact elderly [90, 115].

It is crucial to recognize the potential impact of the many different types of amyloid deposits, as they differ in their associations with clinical symptoms. The most commonly described types are diffuse plaques, which are non-fibrillar and vary in morphology, and dense core deposits [116, 117], which contain fibrillar Aβ and correlate better with clinical severity [118]. Furthermore, dense core plaques can further be characterized by the presence of a neuritic component if the focal Aβ deposit contains tau-positive or dystrophic neurites. Neuritic plaques (NPs) correlate well with clinical severity and are thought to closely associate with neuronal loss or atrophy in AD [119]. For example, in a cohort of individuals with intact cognition, NPs were associated with lower scores on cognition tests [120]. Strikingly, reduced amounts of NPs have also been observed in the superior temporal sulcus in resilient individuals compared to AD patients [91]. Similarly, in resilient donors with similar Braak stages compared to AD patients, reduced amounts of ptau has also been shown in the middle superior gyrus [84] and in NFTs in the temporal gyrus [86]. Also, the progression of ptau could be halted, as in nondemented centenarians very few resilient donors reached Braak stage 5 or a CERAD score for NPs higher than 2 [24]. Similarly, in nondemented donors decreased amounts of Aβ oligomers (oAβ) have been observed in frontal and parietal cortical lysates [77], hippocampal lysates [93] and hippocampal synaptosomes [69], while no differences in the amount of oAβ have been found in the frontal cortex [81]. Furthermore, reduced levels of monomeric and multimeric ptau forms have also been demonstrated in synaptosomes from the superior temporal sulcus [91], temporal sulcus [68] or hippocampus [96] and in the visual cortex [97], indicating that ptau is present before tangle formation in AD and that these levels are lower in resilient donors. oAβ has been shown to induce neurotoxic effects associated with loss of dendritic spines and synaptic function as most synapse-associated Aβ in AD patients consist of soluble oligomeric species and synaptic tau pathology [121–123]. Taken together, the pictures that emerges from these studies is that resilient donors have a reduced amount of AD pathology that correlates better with clinical severity, including NPs, Aβ oligomers and both the progression and load of ptau. Thus, donors classified as resilient based on global AD neuropathological levels are often in fact resistant, or have BM, to specific toxic neuropathological species as they have lower levels than expected of oligomeric species and NPs.

#### Concomitant pathological AD changes

While most studies focused on resilience towards the classic AD neuropathology, i.e. plaques and tangles, more recent studies have included common co-morbid neuropathological changes, such as granulovacuolar degeneration and cerebrovascular disease (CVD) pathologies like infarcts and atherosclerosis. Whereas Lewy bodies (LB), phosphorylated TAR DNA-binding protein 43 (pTDP-43) and hippocampal sclerosis (HS) are usually found in other neurodegenerative diseases, it has been estimated that up to 90% of individuals with AD pathology have other degenerative pathologies [124, 125]. Accumulation of multiple of these concomitant neuropathological changes have been linked to an increased risk of dementia [126]. With respect to resilience, these mixed neuropathologies have been found in cognitive intact individuals [127–130]. Nevertheless, the combinations of AD-associated neuropathology such as LB and TDP-43 differ between individuals. Discoveries of novel pathologies might influence the resilience capability as was recently demonstrated for pTDP-43, which was found to have a lower frequency in resilient than non-resilient individuals while finding no differences in other concomitant AD neuropathology changes [72]. Likewise, others have shown reduced amounts of CVD, pTDP-43, HS and LBs when comparing resilient to non-resilient [90, 131]. Thus, donors classified as resilient in post-mortem studies based on the amount of AD pathology are in fact resistant or have more BM to extensive amounts of comorbid pathology such as pTPD-43, which might also help to maintain cognition in these individuals.

#### Lifestyle factors contribute to both resilience and resistance

The general hypothesis is that resilient individuals maintain cognition despite high amounts of brain pathology through the influence of genetics, early-life experiences, education and lifestyle. In clinical settings focusing on imaging and fluid biomarkers, some studies have shown that proxies like years of education by itself are not attributable to reductions in neuropathological burden [132, 133], while others have demonstrated a reduction in Aβ load [134] or changes in CSF tau or Aβ levels [135, 136]. Studies in post-mortem tissue have substantiated the former results, where education [137], participation in cognitive activities [138] or a combination of these factors [130] have all been associated with a reduced risk of developing dementia independently of pathology. However, a few recent studies suggested that even though these proxies are not associated with classic AD pathology, reductions in cerebrovascular disease like hippocampal sclerosis [139], gross and microscopic cerebral infarcts [140, 141] are seen in individuals with resilience to AD. Thus, cerebrovascular changes may influence resilience capacity. In addition, using EE as an animal model for the influence of factors related to resilience, multiple studies have shown a reduction in the total amount of Aβ or ptau load [40, 56, 142, 143], indicating EE might not only benefits resilience but also resistance, or BM, as there are lower levels of AD-related pathology than expected based on genotype. Others have shown no changes in Aβ deposition after EE while still observing improved cognitive functions [144, 145], suggesting EE can also lead to resilience. This discrepancy between different studies could be attributed to altered parameters such as the model, housing conditions of EE or sex.

In summary, several studies have demonstrated differences in pathology between resilient and AD cases. Reduced amounts of more toxic pathological species such as NPs, oligomers, pathological comorbidities and ptau are likely to contribute to intact cognition in these individuals (Fig. 1). Lifestyle factors may reduce the amount of AD pathology while also protecting against the toxic effects of the pathology, thus influencing both resistance and resilience, or both BM and CR. This further complicates the definition of resilient donors in post-mortem brain tissue as current neuropathological AD staging systems describe the spread of the pathology rather than pathological load. Here, we propose that AD-related pathological changes, which correspond well with cognitive decline, are most important for resilience, such as oligomers, cored plaques and NFTs. To identify mechanisms related to only resilience, both AD and age-related neuropathology should be corrected for in in large community cohorts or should be carefully matched between groups.

### Underlying cellular and molecular mechanisms of resilience

#### Structural and morphological changes in resilience

In AD, structural changes due to atrophy, loss or injury of neurons have previously been established. These include a reduction in total brain weight, cortical neurons, cortical dendrites, spines, synapse densities and reduced brain volume in specific brain regions such as the cerebral cortex, hippocampus or entorhinal cortex [146–149]. Interestingly, some studies have associated larger volumes in hippocampal and cortical regions with lifestyle factors related to resilience, like education [27, 28, 150] or socioeconomic status [151]. However, contradictory results have been reported where no associations with volume changes were shown for a similar score for complex mental activity [152], individuals with a more complex occupation or more leisure activities [153]. In animal models, similar results have been demonstrated where EE lead to increased cortical thickness [154]. Current evidence on morphological and structural changes in resilience is discussed below.

Differences in brain volume on a macroscopic level in relation to resilience have been widely studied. Using PiB PET imaging to visualize Aβ plaque load, increased temporal volumes in cognitively intact individuals with a higher plaque load compared to those with lower plaque load were demonstrated, while opposite effects were found in AD patients [155]. Furthermore, diminished cognitive decline was demonstrated in healthy individuals with a high PiB uptake and hippocampal volume compared to those with hippocampal degeneration [156] or intact hippocampal volume in AD in relation to occupational complexity [157]. Contradictory results have shown cortical thinning in cognitively intact individuals with a high PiB uptake compared to a low PiB uptake in AD vulnerable areas [158]. In addition, low Aβ42 CSF levels, which is often used for diagnostic purposes and is indicative of more Aβ accumulation in the brain, were found in cognitively healthy subjects with increased cortical thinning in the supramarginal gyrus compared to subjects with normal Aβ42 CSF levels [159]. Thus, these individuals remained cognitively intact despite a higher Aβ load in the brain, based on CSF markers, and cortical thinning. Using post-mortem tissue, a decrease in cortical thinning in the PFC of AD patients with a higher cognitive lifestyle score, was demonstrated, but without differences in hippocampal volume [160]. A different study demonstrated a reduction in the number of neurons and cortical thinning in the entorhinal cortex and superior temporal sulcus in AD patients compared resilient individuals [67]. The discrepancies in the results may suggest that resilient individuals remain cognitively intact through different mechanisms than sheer volume in brain structures and can withstand more cortical thinning and reductions in hippocampal volume before clinical manifestations become apparent. Apparently, a large volume is not a prerequisite for the ability to maintain memory function. Thus, it is likely that alterations relevant to resilience are more pronounced on a cellular and molecular level.

One possible compensatory mechanism that has recently been put forward are differences in dendritic spines. In post-mortem tissue of resilient donors, pyramidal neuron dendrites in layers II and III from the dorsolateral prefrontal cortex (DLPFC) had different morphological features. The spine density was higher in these resilient individuals compared to AD patients. In addition, dendritic spines in resilient donors were longer compared to both AD patients and age-matched controls [70]. Subsequent studies have shown positive correlations between lower MMSE scores and a reduced spine head diameter [161]. Synaptopodin-labeled dendritic spines were preserved in resilient individuals compared to AD patients in the same brain region [162]. Furthermore, correlations were found for Aβ plaques and NFTs with increased spine length, reduced thin spine head diameter and increased density of filopodia, which is considered to be a precursor of dendritic spines [163]. Likewise, the protein neuritin (NRN1) was identified as a hub protein related to synaptic changes in resilient donors based on a network analysis of proteomic data. NRN1 was able to rescue Aβ-induced dendritic spine loss in vitro [79]. Lastly, in animal models exposed to EE, or trained for spatial or associative learning tasks, increased density of spines have been found [164, 165]. Together, these results indicate that dendritic spines might change in morphology in the presence of AD neuropathology in resilient individuals compared to both controls and AD donors to maintain their function and/or that in demented AD patients particular types of spines are selectively lost. As dendritic spines in resilient donors have a different morphology to both control and AD donors, it is likely that this molecular mechanism can be attributed to resilience or CR.

#### Neuronal and network activity

In the last decades, it has become more apparent that increased neuronal activity decreases neuronal vulnerability towards AD changes, a phenomenon that was originally paraphrased as ‘use it or lose it’ [5]. For example, the size of the Golgi apparatus, as a measure of metabolic activity, has been demonstrated to increase in the nucleus basalis of Meynert in the early Braak stages [166]. Additionally, changes in gene expression involved in synaptic activity, plasticity and energy metabolism were demonstrated in the prefrontal cortex in early AD, i.e. Braak II-III [167]. These studies demonstrate possible compensatory mechanisms in early, preclinical AD stages that might counteract the neuropathological changes to postpone cognitive impairment. Furthermore, changes in network activity has been associated with resilience in several clinical studies using fMRI, in which specific networks, such as the left frontal cortex connectivity, were more active in resilient individuals [85, 168, 169]. Likewise, maintenance of resting-state functional connectivity was measured in the hippocampus in aged rats with intact learning abilities, similar to younger animals [170]. This could be indicative of maintenance of network connectivity that might protect against age-related cognitive decline. Likewise, preventing network hyperexcitability in AD animal models reduced Aβ deposition and rescued memory deficits [171, 172]. Current studies substantiating claims on neuronal activity on a molecular level related to resilience are sparse, albeit hypertrophy of neuronal cell bodies has been shown in resilient individuals in the anterior and posterior cingulate gyrus, CA1 and primary visual cortex [80, 103, 173], which could be a compensatory mechanism, a sign of increase in activity or an indication of increased metabolism in reaction to AD pathology. More recently, the transcription factor myocyte enhancer factor-2 (MEF2) was identified as possible regulator of neuronal activity in resilience, as it was upregulated after EE, a knock-out of MEF2 resulted in cognitive impairments and overexcitability and overexpression in a tauopathy model (P301S) improved cognition [66]. Furthermore, MEF2 expression was increased in a subpopulation of excitatory neurons in post-mortem tissue of resilient donors compared to AD patients. In addition, in a large ageing cohort including resilient and AD donors, the relative abundance of inhibitory neurons tended to be higher in resilient donors, which was specifically larger in the reelin (RELN) positive inhibitory neurons of the LAMP5 subtype [88]. Reelin signaling is important for neuronal development, cytoskeleton regulation and synaptic plasticity [174]. Interestingly, alterations in *RELN* expression have been demonstrated in AD [175], which has been associated, among others, with increases in ptau and synaptic dysfunction [174]. Hence, this subtype of inhibitory neuros might protect against the toxic effects of AD in resilient donors.

#### Synaptic changes

Other synaptic changes in resilient individuals include preservation of synapses and synaptic signaling. In resilient individuals, a similar number of synaptophysin (SYP) labeled presynaptic terminals compared to healthy controls was found, while a decrease of SYP was observed in AD patients in the medial frontal gyrus [65]. Similar results were obtained in a different cohort, which showed a striking preservation of neurons, synaptic markers and axonal geometry in the superior temporal sulcus of individuals with a mismatch between post-mortem AD pathology and ante-mortem cognition [91]. These individuals, with a similar amount of AD pathology as AD patients, had similar protein levels of postsynaptic density 95 (PSD-95) and SYP compared to healthy controls, while these levels were reduced in AD patients in homogenate frozen tissue samples of the superior temporal sulcus. Others have also demonstrated decreased SYP and synaptopodin levels in the middle frontal gyrus in demented compared to non-demented cases with a Braak score higher than III [65, 105], whereas no differences were observed in protein levels of PSD-95 and growth-associated protein 43 (GAP-43) [105]. Importantly, the authors showed a correlation between SYP protein levels and the ante-mortem MMSE score. In contrast, in a different cohort no differences in the levels of SYP were demonstrated in the frontal cortex between resilient individuals and AD patients [87]. Combined, these studies suggest that resilient individuals maintain their synapse numbers despite the presence of AD-pathology.

Besides changes in protein levels in synapses, altered gene-expression related to pre- and post-synaptic terminals have also been found using a network analysis in the posterior cingulate cortex of cognitively intact donors with Braak stages III/IV and I/II [176]. These included, amongst others, glutamate ionotropic receptor NMDA type subunit 2A (*GRIN2A*) and solute carrier family 17 member 6 (*SLC17A6*). Furthermore, using multiple cohorts to compare AD, resilient and control donors, several networks related to neuronal and synaptic proteins were found based on proteomics data from the DLPFC [82, 94]. These networks were significantly downregulated in AD compared to controls while resilient donors, although not significant, were also downregulated. Even though the amount of tau pathology was lower in the resilient donors, these results point towards changes in neuronal and synaptic proteins prior to symptom onset. Finally, in a different cohort, enrichments for serotonin and neuronal signalling were found using proteomics in the parietal cortex in both resilient and control donors compared to AD patients [74].

Interestingly, preservation of synaptic markers in individuals with a high pathology load and no cognitive impairment has also been shown in the hippocampus, although results vary between studies. Whereas no significant loss of neurons in the hippocampus was observed in resilient subjects, only protein levels of SYP were maintained in these individuals, while other proteins concentrations were reduced similar to AD patients, like VAMP and synaptotagmin [102]. Recently, besides increased levels of SYP, also higher levels of neurofilament light chain (NFL-L) and Park5 were found in the hippocampus of resilient donors compared to AD patients, which might indicate healthier axons, dendrites and synapses [98]. In contrast, in a different cohort using Western blot, only the postsynaptic protein PSD-95 was preserved in resilient individuals, while all other pre- and postsynaptic proteins, such as Debrin, synapsin-1 (SYN1) or synapse associated protein 97 (SAP-97), were reduced similar to AD individuals compared to controls with low pathology and normal cognition [93]. In addition, by isolating hippocampal postsynaptic densities, fifteen proteins were found that were uniquely different in resilient individuals compared to AD patients and healthy controls [100]. These included calcium/calmodulin-dependent protein kinase type II subunit alpha (CAMKIIα), keratin type 1 (*KRT10*), actin 2 and SYN1, which are crucial for activity-dependent synaptic remodeling [177] and are mainly involved in cytoskeletal function. However, the authors were unable to confirm the changes of a subset of proteins in hippocampal tissue from a different cohort of patients. Futhermore, increased protein levels of glutamate receptors such as AMPA Type Subunit 1 (GluA1) and metabortopic glutamate receptor 3 (mGluR3) have been demonstrated in hippocampal lysates of resilient 5xFAD mice, identifyed by comparing learners and non learners [89]. Increased levels of GluA1 in learners is not suprising, since the insertion of GluA1-containing AMPA receptors into synapses is required for learning [178]. Notably, this enrichment of GluA1 at synapses likely comes at the expense of GluA3-containing AMPA receptors, which are abundant in inactive synapses [179]. Because the presence of GluA3 is sufficient to make synapses vulnerable to Aβ [62], learning may potentially render synapses resilient to Aβ through a change in AMPA receptor subunit composition. In AD-BDX animals that maintained cognition despite increased Aβ levels, increased gene expression was shown in the GABA transporter *SLC6A13* [180]. Furthermore, by using cognitive training as an attempt to delay cognitive decline via the morris water maze, increased levels of PSD-93, PSD-95 and SYP were found in the PR5 animal model, which overexpresses the longest human tau isoform together with the P301L mutation [181]. Finally, increased levels of SYP, PSD-95 and synaptic plasticity have been found in WT animals and in different AD animal models in the hippocampus after EE [182, 183]. Interestingly, increased levels of PSD-95 at synapses were shown to protect againts synaptic deficits induced by Aβ [184], which could explain the preserved cognition in these models.

**Fig. 2:**
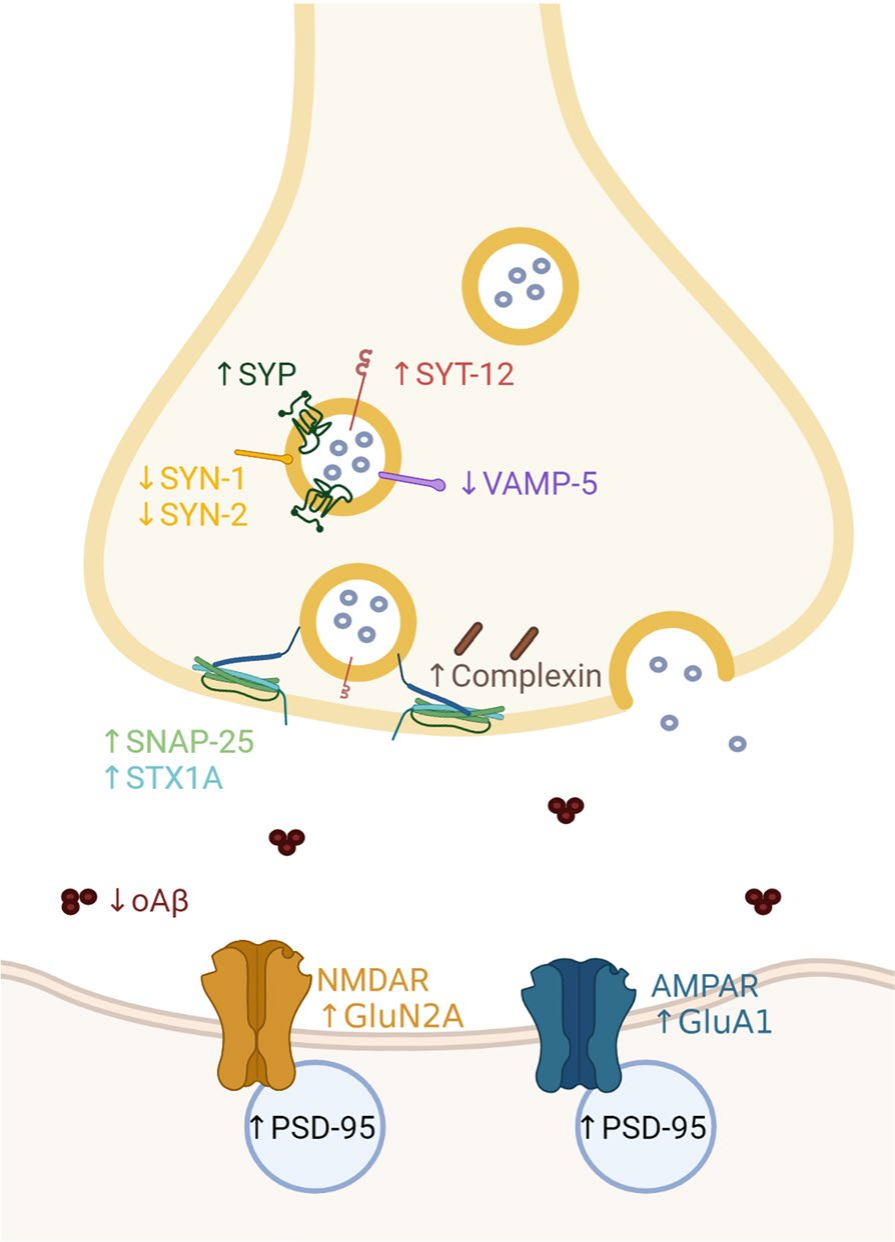
Synaptic changes in resilient individuals compared to AD patients. Simplified overview of synaptic changes in resilient donors compared to AD patients. The most pronounced changes in the resilient synapse compared to the AD situation in cortical regions are alterations in the SNARE proteins, reduced amounts of oAβ and increased levels of PSD-95. More sparse is the evidence for alterations in receptors and their subunits, such as AMPAR and NDMAR, which has only been found in animal models. Alterations in synaptic changes in hippocampal regions is less evident than in cortical regions. Abbreviations: AMPAR; α-amino-3-hydroxy-5-methyl-4-isoxazolepropionic acid (AMPA) receptor, GluA1; AMPAR subunit 1, GluN2A; NMDAR subunit type 2A, NDMAR; N-methyl-D-aspartate receptor, oAβ; oligomeric amyloid beta, PSD95; postsynaptic density protein 95, SNAP25; synaptosome-associated protein of 25 kDa, STX1A; Syntaxin 1A, SYN; synapsin. SYP; synaptophysin, SYT-12; synaptotagmin 12, VAMP-5; vesicle-associated membrane protein 5

Several studies have found differences in synaptic proteins involved in vesicle signaling and fusion, such as the soluble N-ethylmaleimide-sensitive-factor attachment protein receptor (SNARE) proteins (Fig. 2). Higher protein levels of complexin I and II, synaptosome-associated protein of 25 kDa (SNAP25), syntaxin-1A and 1B (STX1A/B) and vesicle-associated membrane proteins 1/2 (VAMP1/2), have previously been associated with cognition, after correcting for the amount of AD pathology in post-mortem tissue, in different cortical regions susceptible to AD, including the hippocampus [185]. Subsequent studies have further elucidated the putative role of complexin in AD, as complexin I levels were most associated with cognition in Braak 0-II while lower levels of complexin II were associated with a reduction in cognition in Braak III-VI after correcting for pathology and synapse density [186]. More recently, using proteomics in an autopsy community-based cohort, eight cortical proteins were associated with resilience, which were identified in individuals that maintained cognition over time after controlling for age-related neuropathology. These, amongst others, included rabphilin 3A (RPH3A), complexin 1 and NRN1, which are related to vesicle signaling and neurite outgrowth [99]. In addition, using targeted proteomics in a larger community sample including AD and resilient samples, higher levels of *STX1A,* synaptotagmin 12 (SYT12), *SNAP25* and syntaxin binding protein 1 (STXBP1) were associated with better cognitive performance, while STXBP4, STX7 and SYN2 correlated with a worse cognition [92]. These findings have been substantiated by the same researchers after correcting for cognitive decline and AD pathology, which pointed to increased STX1A, SNAP25, SYT12 and decreased SYN2, vesicle associated membrane protein 5 (VAMP5), vesicle amine transport 1 (VAT1) and SLC6A12 in resilience [101]. On a transcriptomic level, reduced amounts of, amongst others, *SNAP25*, *STX1A*, *STX17*, *SYN1* and *VAMP1-4*, have been found in resilient donors, similar to AD, in multiple brain regions, including hippocampus, entorhinal cortex, frontal and temporal cortex [187]. This shows that there is not a clear overlap between protein and RNA levels while more importantly, the amount of resilient individuals included in Liang et al., was low. Most of these studies point to differences in the SNARE proteins in resilient donors, which might help them in maintaining synaptic functioning.

Bjorklund et al., 2012 reported lower total Zn^2+^ levels and lower levels of its synaptic vesicle transporter zinc transporter 3 (ZnT3) in postsynaptic hippocampal factions in resilient versus AD donors, while similar levels of synaptic vesicle Zn^2+^ were observed between both groups. The regulation of Zn^2+^ has previously been linked to synaptic targeting of oAβ [188–190], suggesting that that Zn^2+^ regulation via ZnT3 impairs this mechanism in resilient individuals. Interestingly, multiple studies have made the observation of reduced amounts of oAβ in either tissue lysates or synaptic fractions in resilient donors [77, 93]. Additionally, the authors found that resilient individuals maintained cyclic AMP (cAMP)-response element binding protein (CREB) (pCREB) levels, a transcription factor vital for synaptic plasticity and memory function [191]. Finally, as oAβ was shown to bind many other targets in the synaptic cleft [192], there may be other mechanisms that contribute to the clearance of these oligomers in resilient individuals.

In summary, both from post-mortem human tissue and from animal models the notion that maintaining cognition can be achieved by altering synaptic signaling is evident. Overall the data support the hypothesis that compared to demented AD patients or cognitively impaired transgenic animals, resilient individuals or animals both have, often similar to their respective controls, preservation of neurons and synapses accompanied by altered synaptic signaling. It is likely that these molecular and cellular processes can be attributed to resilience or CR.

#### Neurogenesis and growth factors

Adult human neurogenesis (AHN) occurs predominantly in the dentate gyrus and subventricular zone located in the walls of the lateral brain ventricles [193, 194]. Studies on AHN so far have demonstrated mixed results, although it is generally believed that neurogenesis is active throughout adulthood and declines with age [195]. Cellular markers used to study neurogenesis are derived from animal models and include doublecortin (DCX), proliferating cell nuclear antigen (PCNA) or SRY-Box Transcription Factor 2 (SOX2). Whereas they are well conserved across different species, they suffer from several caveats including sensitivities to post-mortem delays, fixation time, expression of multiple markers at a given time and lack of validation in human samples [196, 197]. Recent studies have indicated that higher numbers of neuroblasts, measured with DCX and PCNA, are associated with better cognitive status and that adult human neurogenesis is reduced in the later stages of AD [196, 198, 199]. Similarly, more immature neurons have been identified as neurogenic populations from single-cell sequencing approaches in post-mortem hippocampal tissue of aged individuals compared to AD patients [194, 200]. Thus, it might be possible that cognitive impairment in AD can be ameliorated by diminishing the reduction of hippocampal neurogenesis. Remarkably, it has been found that neurogenesis, measured with the transcription factor SOX2, might be increased in resilient individuals, as populations of neuronal stem cells were preserved in the dentate gyrus in these individuals, similar to controls, while these were lower in AD patients [71]. The same authors showed that increased amounts of neurons did not correlate with increased cognition, hypothesizing that other mechanisms of neuronal stem cells might play a role. Nevertheless, the results were based on just 4 resilient donors. The possible preservation of neurogenesis in resilient individuals remains to be further elucidated with markers that study neurogenesis directly instead of precursor stages.

A possible mechanism through which neuronal stem cells may contribute to maintaining cognition, aside from adding new neuronal populations, is through the release of exosomes, often filled with microRNAs (miRNAs). Interestingly, miRNA-485, miRNA-4723 and miRNA-149 were differentially expressed in the hippocampus or frontal cortex in AD compared to healthy controls or resilient individuals [201]. Administration of exosomes isolated from neuronal stem cell cultures, which contained the same miRNAs, have shown to ameliorate cognitive deficits in vivo in mice and protect against synaptic deficits in vitro [201, 202]. Thus, it can be hypothesized that the increased levels of miRNA’s that regulate synaptic genes find their origin in exosomes released from neuronal stem cells. Furthermore, miRNAs from exosomes have previously been linked to aging, synaptic plasticity [203] and neuroprotection [204]. However, exosomes have also been shown to be involved in the propagation of tau in AD [205, 206]. Evidently, the role of extracellular vesicles in AD progression and in resilience is an exciting topic for further study.

Higher serum brain derived neurotrophic factor (BDNF) levels have been linked to a reduced risk of developing dementia [207] and with a slower cognitive decline with the strongest effects in individuals with higher amounts of AD pathology [208]. Importantly, this effect remained significant after accounting for AD neuropathology. BDNF could possibly contribute to resilience as it has previously been associated with neurogenesis [209], synaptic plasticity [210] and dendritic density [211]. In line with this, administration of BDNF restores learning and memory in different mouse models of AD and in aged rats [212]. In addition, social interactions and environmental enrichment improve memory-deficits through BDNF-dependent hippocampal neurogenesis [209, 213]. Furthermore, a recent small scale intervention study demonstrated an increase in serum BDNF levels in females with mild cognitive impairment (MCI) after eight weeks of mental training [214]. Nevertheless, no differences in BNDF levels from frontal tissue homogenate were found in resilient individuals, aged 90 years or older, compared to age-matched AD patients [87].

Although the effects of BDNF in resilient individuals remain elusive, differences have been found in other neurotrophic factors. Increased levels of vascular endothelial growth factor A (VEGFa), nerve growth factor inducible (VGF), fibroblast growth factor 2 (FGF2) and platelet-derived growth factor (PDGF) and decreased levels of pigment epithelium-derived factor (PEDF), have been found in resilient donors compared to AD patients [67, 87, 101]. Furthermore, numerous studies have demonstrated increased levels of growth factors in the hippocampus of animals after EE, such as nerve growth factor (NGF), VEGF or BDNF [215–217]. VEGF, FGF2 and PEDF are involved, amongst other roles, in neurogenesis and plasticity [reviewed in: [218–222]. Furthermore, FGF2 reduces inflammation and amyloid deposition in the hippocampus [223, 224] while VEGFa is also involved in angiogenesis, neural migration and protection and blood–brain-barrier integrity [221]. NGF regulates the functional state of cholinergic neurons in the basal forebrain, which has been demonstrated via retrograde transport from the hippocampus and influences learning and memory. Overexpression of growth factors, including FGF2, PDGF, VEGF or NGF have been linked to improvements in hippocampal functioning in AD mouse models [225, 226]. Interestingly, in light of the results from epidemiological studies and the promising role of growth factors in animal models, NGF has been tested in humans [227], whereas a clinical study for BDNF is underway.

Taken together, these results indicate that environmental influences, in particular in animal models, can increase the amount of growth factors, which is likely to contribute to cognition. It is therefore conceivable that some of these growth factors also play a role in resilience to AD. Maintaining levels of growth factors and the possible maintenance of neurogenesis in resilient donors could be attributed to BM, as the levels remain similar to controls.

#### Glial cells and inflammation

In recent years, the role of the immune system in the pathophysiology of AD has received more and more attention. Neuroinflammation, characterized by reactive astrogliosis, microgliosis and release of inflammatory cytokines, has been associated with disease progression and cognitive decline [43, 228]. Initially, the reaction of microglia and astrocyte towards AD pathology is beneficial as these cells are involved in Aβ clearance. However, over the course of the disease, activated microglia shift to a more pathological state in which they release pro-inflammatory mediators, such as interleukin (IL)-1β, IL-6, and tumor necrosis factor-α (TNF-α). Genome-wide association studies (GWAS) have identified numerous genetic variants associated with microglia in AD, which could increase the risk to develop AD. The most studied variant is in triggering receptor expressed on myeloid cells 2 (TREM2). The TREM2 variants have been hypothesized to hamper the ability to phagocytose Aβ [229]. In addition, multiple studies have shown an altered state of microglia in AD, as indicated by markers associated with microglial activation, including HLA-DR and CD86 [230, 231], or by an altered transcriptomic profile, such as disease-associated microglia (DAM), or based on morphological changes [232]. Similarly, reactive astrogliosis has also been found to accumulate around AD pathology [233, 234], which is characterized by increased expression of glial fibrillary acidic protein (GFAP), morphological alterations and functional changes [235] and is inversely associated with cognition [236]. While activated astrocytes provide neuroprotection via release of neurotrophic factors and have a role in the degradation of Aβ [237], they also play a role in neuroinflammation.

Remarkably, multiple studies have demonstrated a lower density and a different morphology of glial cells in post-mortem human brain tissue in resilient individuals compared to AD patients. Increased levels of markers related to glial cells such as TREM2 and tyrosine kinase binding protein (TYROBP) have been found in the temporal cortex [238], and complement C5b-9 and HLA-DR in the entorhinal cortex and frontal gyrus superior [239] and HLA-DR, DP and DQ sub-regions in the hippocampus [240] in AD patients versus resilient donors with a high Aβ load and healthy controls. Others have shown different microglial states in AD donors compared to both resilient donors with similar amounts of both Aβ and tau pathology and control donors based on the marker CD68 in the entorhinal cortex and superior temporal sulcus [67, 91]. Similarly, increased CD68 and a trend toward increased HLA-DR and decreased TM119 and P2RY12 has been shown in both the temporal pole and visual cortex in AD patients compared to resilient and control donors, demonstrating that these changes already become apparent in the visual cortex before the presence of AD pathology [53]. Finally, increased CD68 has also been demonstrated in the environment of NFT-bearing neurons in AD donors compared to resilient individuals in the CA1 region of the hippocampus [98]. Others have shown similar levels of HLA-DR in the middle frontal gyrus or superior and middle temporal gyri between AD cases and resilient donors with only a high Aβ load [241], making it impossible to determine whether these individuals are resilient or would have developed dementia in time. Similar levels of microglial activation between AD patients and resilient donors with only high amounts of Aβ pathology have also been demonstrated in the precuneus with the marker CD36 [242]. Very recently, increased levels of Iba1, TREM2, TYROBP and CD68 were found near plaques in the frontal cortex when comparing resilient with AD donors, indicating not only a higher number but also more hyperactive microglia around plaques in resilient donors [78]. The authors hypothesized that these results indicate a phagocytic microglial subpopulation in resilient versus demented AD individuals, as more healthy synapses were observed near plaques in resilient donors, based on MAP2 or βIII Tubulin as axonal or dendritic markers and the amount of phosphatidylserine from isolated synaptosomes. Phosphatidylserine has been hypothesized to be a neuronal “eat-me” marker for microglial synaptic pruning. In animal models, a striking observation was made after crossing either APP/PS1 or PS19 (harboring MAPT^p301s^) mice with animals with *TYROBP* knockout animals*.* Both models showed improvement in learning behavior and synaptic signaling compared to transgenic animals with WT *TYROBP* while having similar amounts of AD pathology [243, 244]. In addition, the knockout also reduced microglia recruitment around plaques, reduced levels of complement protein C1q and reduced expression of genes related to the switch in phenotype from homeostatic microglia to DAM. One hypothesis that could explain the discrepancy between the results of post-mortem human tissue and animal models with respect to *TYROBP* functioning is that in resilient donors microglia become efficient in clearing Aβ without adapting a DAM-like phenotype or developing senescence over time, while in animal models microglia are adapting this DAM-like phenotype. Furthermore, lower expression of susceptibility genes for AD related to microglial function, such as low affinity immunoglobulin gamma Fc region receptor II-b (*Fcgr2b*), cathepsin H (*Ctsh*), hematopoietic cell-specific Lyn substrate 1 (*Hcls1*) or integrin subunit beta 2 (*Itgb2*), have been found in resilient AD-BDX mice, identified by comparing animals with intact and impaired cognition with similar amounts of Aβ pathology [180]. Interestingly, decreased expression levels of the inflammatory pathway of NOD-, LRR- and pyrin domain-containing 3 (NLRP3) inflammasome, caspase-1 and IL-1B were demonstrated after a behavioral training paradigm as a model to stimulate learning experiences in PR5 animals [181], which highlights the possibility that lifelong factors in resilient donors might cause a reduction in the NLRP3 inflammasome. Activation of the NLRP3 inflammasome has been demonstrated in both AD patients and high pathology controls in the medial temporal lobe, indicating that inflammation as a reaction to AD pathology is also present before clinical symptoms appear. Nevertheless, as the amount of pathology varied in the high pathological controls, it remains difficult to determine if these are resilient donors [245].

Several studies have implicated a role for astrocytes in resilience. In post-mortem tissue of resilient individuals, similar GFAP levels have been found compared to age-matched controls while higher amounts were found in AD patients in the superior temporal sulcus [91], entorhinal cortex [67], medial frontal or superior and medial temporal gyri [241] and temporal pole and visual cortex [53]. Also, in the environment of NFT-bearing neurons an increase of GFAP has been found in AD compared to resilient donors in the hippocampus [98]. Others have found thicker and longer processes in astrocytes in resilient donors compared to both AD and control donors and increased Glutamate transporter-1 (GLT-1) expression in astrocytes in resilient and control donors compared to AD patients in the entorhinal cortex [83]. GLT-1 is the main glutamate transporter in the brain and a reduction has been associated with cognitive impairment in AD. Whereas, increased densities of GFAP has been found in the medial frontal gyrus cortex in resilient individuals compared to AD-patients [65], no differences were found in GFAP protein levels using ELISA between resilient individuals and AD patients in the frontal lobe [87]. The same study also demonstrated increased levels of S100B in AD patients, which is a molecule secreted by astrocytes and is both associated with trophic and toxic effects, such as increased pro-inflammatory cytokines and influxes of Aβ at the blood–brain barrier via the S100B/RAGE pathway.

Only a handful of studies have used omics technologies in resilient post-mortem tissue to investigate different glial states. Using a network approach of co-expressed proteins based on proteomics data, several networks related to glial cells were identified that were significantly activated in AD compared to control, while high pathology controls with similar levels of Aβ, but reduced levels of ptau, did not differ between both conditions [82, 94]. Likewise, different cellular communities, or cell states that correlate with each other, were identified in AD and resilient samples. These cellular communities correlated well with demented individuals and a high tau burden, while others were associated with unimpaired resilient individuals with a lower tau burden [73]. This would suggest that different cell states, including different microglia and astrocyte states, may be important mediators of cognitive decline. Finally, plaque-induced genes (PIGs) have been identified as a local response to Aβ, concerning genes of the complement system, oxidative stress, lysosomes, and inflammation, which are mainly enriched in glial cells near Aβ plaques [246]. As it is hypothesized that resilient individuals are able to withstand more Aβ plaques or are able to more effectively clear Aβ plaques, it might be possible that the glial cells have a different reaction to the plaques by having a lower expression of these PIGs.

In AD, microglia and astrocytes are conceivably the primary source of cytokines, which play an important role in proinflammatory and anti-inflammatory processes. Increased synthesis of the pro-inflammatory cytokines TNF-α, IFN-γ, IL-1β, IL-6, IL-18 and their cognate receptors have been found in AD [247]. A recent study has indicated that resilient individuals have distinct profiles of cytokines in the entorhinal cortex and superior temporal sulcus. Individuals that remained cognitively intact despite a high or intermediate amount of AD pathology had lower levels of the pro-inflammatory cytokines IL-1β, IL-6, IL-13, IL-4 or IL-6, IL-10, IP-10, respectively, compared to AD patients and controls [67]. Interestingly, the authors demonstrated a reduction in expression of chemokines associated with microglial recruitment, such as MCP-1 and MIP-1α in resilient individuals compared to AD patients. Similarly, a reduced amount of CD8 + T cells have been found in both the hippocampus and PFC in resilient individuals [240]. It could be postulated that glial cells in AD patients are not able to clear Aβ plaques, which results in a more pathological phenotype, in which they release more pro-inflammatory cytokines. In the resilient situation, these glial cells might be able to clear Aβ better and are not obtaining such a pathological phenotype resulting in a different cytokine profile.

To summarize, most studies showed reduced amounts of activated glial cells associated with AD pathology, activated microglia with effective phagocytotic capacities and possibly a different cytokine profile. Less neuroinflammation in these donors could prevent aberrations in microglia homeostatic functions like surveillance, synaptic pruning and plasticity and more efficient clearance of Aβ [248]. Thus, it can be hypothesized that the differences between glial states between resilient individuals and AD patients contribute to both resilience or CR, as they might stay in more homeostatic states despite the presence of AD pathology, and resistance or BM, as they can effectively clear pathology, lowering the amounts of AD pathology. In addition, glial cells have been shown to be influenced by lifestyle factors as they are sensitive to changes in the enviroment [249, 250]. For example, in AD animal models, environmental influences via EE were shown to prevent astroglial pathological changes [57, 251]. As a recent animal study indicated that newly formed neurons do not integrate in the dentate gyrus in AD due to changes in the microenvironment [34], maintenance of homeostatic functions of microglia might preserve the microenvironment and thereby maintain adult neurogenesis. So far, studies on glial cells in resilience have relied on histological activation markers, limiting the classification of different microglial or astroglial subtypes and thereby hampering the interpretation of these results [252, 253]. Ultimately, a whole range of phenotypic remodeling may occur in these cells, some of which are related to neuroprotection while other changes are pathological. It is vital to validate whether different activated microglia or astroglia subtypes might protect against or exacerbate the pathophysiology of AD, and to investigate if previously identified DAM or PIGs are present in resilient donors by using single-cell approaches.

#### Mitochondrial changes

To date, many studies have shown mitochondrial dysfunction in the brain of AD patients and impaired energy metabolism is an early and consistent feature in AD [254]. Using fluoro-2-deoxyglucose positron-emission tomography (FDG-PET), a decline in glucose utilization has been found in multiple brain regions in AD [255]. As glucose hypometabolism has been generally interpreted as impaired energy metabolism via, amongst others, oxidative phosphorylation, changes in glucose utilization have also been implicated to involve mitochondrial dysfunction. Interestingly, Arenaza-Urquijo et al. [64] showed maintenance of glucose metabolism in resilient individuals by using FDG-PET in the bilateral anterior-mid cingulate, medial prefrontal and anterior temporal lobes. The resilient individuals were identified based on intact cognition, Aβ load measured by PET PiB and cortical thickness. Consistent with the findings of impaired energy metabolism in AD and its possible maintenance in resilience, multiple studies have identified changes in mitochondrial related metabolic pathways in AD [256, 257]. Remarkably, in post-mortem brain tissue using proteomics, an decrease in mitochondrial subunits was found after a network analysis in the DLPFC and precuneus of AD patients compared to both resilient and control donors [82, 94]. On a proteomic level, multiple mitochondrial subunits were associated with resilience in the dorsolateral prefrontal cortex after correcting for cognitive decline and AD pathology in a large community-based cohort [101]. Similarly, using protein lysates enriched for synaptic fractions from the parietal cortex, enrichments of mitochondrial proteins were present in resilient donors compared to AD patients [74]. Besides post-mortem tissue, differences in protein abundance in cognitively intact 5xFAD mice compared to impaired animals included proteins related to mitochondrial functioning [89]. Together, these results point to maintenance of mitochondrial function in resilient and control donors opposed to the apparent mitochondrial dysfunction in AD patients.

#### Repair mechanisms and cellular health

If mitochondrial dysfunction might be lower in resilient donors, reduction in oxidative stress and resulting DNA damage might be decreased. Increased DNA damage as a result of oxidative stress has been established to play a role in the pathogenesis of AD [258]. For instance, proteins that are related to high cellular stress and energy demands that are upregulated in response to oxidative stress, such as, PTEN induced kinase 1 (PINK1) and NADP + dependent isocitrate dehydrogenase 1 (IDH1), were reduced in the microenvironment of NFT-bearing neurons in the hippocampus of post-mortem tissue from resilient donors compared with AD patients [98], indicating that resilient donors might have reduced levels of oxidative stress. Furthermore, aberrant levels of Ki-67 were found in AD compared to the resilient donors, from which the authors hypothesized that in the resilient NFT-bearing donors there was less entry into a senescence phenotype. However, in a different cohort, protein markers for oxidative stress, such as protein carbonyls PC, 4-hydroxynonenal 4-HNE and 3-nitrotyrosine, were upregulated in hippocampi of both high pathology controls and donors with MCI in comparison to low pathology controls [93]. In addition, AD donors showed high levels of oxidative DNA damage compared to control, while this was lower in the resilient compared to the AD donors. In the same donors significant reductions were observed in DNA repair in AD patients compared to both control and resilient donors [95]. Likewise, more DNA damage in AD patients compared to control, but not resilient donors, has been substantiated with the marker YH2AX in the temporal and between AD patients and both control and resilient donors in the visual cortex [53]. Hence, resilient individuals might maintain cognition by reducing DNA damage, which might increases as pathology progresses. Other molecular mechanisms related to cellular health and maintenance of repair include the ubiquitin–proteasomal pathway, which was shown to be differentially regulated in multiple cortical brain regions, in both resilient and AD compared to control donors [187], while more recently ubiquitin-like modifier activating enzyme 1 (UBA1) was shown to be associated with resilience after correcting for pathological comorbidities [99]. Lastly, higher CD47 levels were linked to resilience in synaptosomes [259] or in proteomics in a community sample after accounting for cognitive decline and AD pathology [101]. CD47 is a cell surface ligand, acting as a "don't eat me" signal to prevent phagocytosis. Thus, it can be hypothesized that cellular health and DNA repair mechanisms are maintained in resilience to AD. Some mechanisms are likely related to BM, such as the possible absence of oxidative stress, reduced levels of cell senescence, and increased CD47. On the other hand, UBA1 has been associated with a range of different processes ranging from protein degradation to neuronal function [260] and thus cannot be assigned to only resilience or resistance, or CR or BM.

#### Genetic factors

Over recent years it has become evident that genetics play a large role in the development of AD. It has been estimated that the heritability of AD lies between 50–80% [261], suggesting that susceptibility or resilience to AD is partly determined by genetic factors. Specific single-nucleotide polymorphism (SNPs) that increase the risk of AD could be absent in resilient individuals, those who increase cognition might be more present and finally novel SNPs from GWAS studies in resilient donors could point to novel mechanisms. Examples of these SNPs will be discussed in the next section.

LOAD has been associated with multiple common variants with a low effect size [262, 263], with apolipoprotein E (ApoE) as the strongest genetic risk locus. ApoE proteins form lipoprotein particles that are involved in lipid transport and contribute to both clearance and deposition of Aβ peptides and has been associated with longevity, depending on the isoform. ApoE4 has been shown to reduce the clearance and increase the deposition of Aβ in plaques and to decrease metabolic activity compared to ApoE3 [264], while ApoE2 has been associated with a reduced risk of AD [265]. Carrying the ApoE4 allele would therefore not help acquiring resilience. Nevertheless, several studies have shown that lifestyle factors such as education, leisure activities or maintaining vascular health can still reduce the risk of dementia in ApoE4 carriers [266–269]. Besides genetic variants in ApoE, other studies have shown that certain SNPs associated with AD might be less prevalent in resilient donors. For example, one of the highest risk factors from previously performed GWAS studies in AD are variants in TREM2. While there are no studies to date demonstrating a reduced prevalence of SNPs in TREM2 in resilient donors, it is likely that resilient donors have a reduced genetic risk for AD and thus lack SNPs related to AD. More TREM2 was recently demonstrated around plaques in post-mortem tissue of resilient donors [78]. Furthermore, the absence of other well-known SNPs related to AD like clusterin *(CLU)*, phosphatidylinositol binding clathrin assembly protein *(PICALM),* manganese superoxide dismutase *(MnSOD),* ATP binding cassette subfamily A member 7 *(ABCA7)* or microtubule-associated protein tau *(MAPT)* have been linked to better memory function in individuals that were 90 + years old [270, 271] or in resilient donors [272].

Besides genetic variants that may increase susceptibility to AD, several studies have identified individual SNPs that may promote cognitive functioning in adults. For example, variants in *KLOHTO* or *RAB10* are associated with enhanced cognition [273, 274] or intact cognition in individuals with a high genetic risk of AD [275]. The *BDNF* Val66Met polymorphism impairs the expression of *BDNF* and the absence of this SNP has been associated with individuals labeled as resilient [276, 277]. Another SNP that has been found in non-demented elderly with a high compared to a low NFT burden is the glycoprotein reelin (*RELN*) [278]. Furthermore, a gain of function SNP in *RELN* was recently identified in a resilient individual to fAD, which maintained cognition up to three decades after the expected onset of symptoms [110]. More recently, an increased proportion of RLEN-positive inhibitory neurons of the LAMP5 subclass was found in post-mortem tissue of resilient donors [88]. The increased expression of reelin in resilient individuals in specific cell types or through polymorphisms in *RELN* might protect against AD pathology and its toxic effects. Finally, variants in phospholipase C gamma 2 (*PLGC2*) have been associated with longevity [279], which is enriched in microglia and hypothesized to be in the same signaling pathway as *TREM2* [280].

Other studies have performed gene-wide association studies (GWAS) on resilient individuals by characterizing individuals based on their global cognitive performance after controlling for demographics and neuropathology [281]. These variants included unc-5 netrin receptor C *(UNC5C)*, ectodermal-neural cortex 1 *(ENC1)*, and transmembrane protein 106B *(TMEM106B),* which have been linked to increased risk of LOAD and susceptibility to neuronal cell death [282], neuroprotective mechanisms [283] and reduced risk for TDP-43 inclusions [284], respectively. Other researchers identified different genetic variations associated with resilience in individuals whose cognitive functioning was better than predicted based on neuroimaging [285]. Only a single SNP in ribonuclease A family member 13 (*RNASE13)* was associated with a better performance that was highly associated with pathways related to spines, presynaptic membranes, and postsynaptic densities [286, 287]. While these previous studies all had limited sample sizes, this problem was recently tackled by combining recent publicly available genetic and in vivo imaging data from a clinical trial and three longitudinal cohort studies of AD [75]. A SNP associated with resilience was identified in ATPase phospholipid transporting 8B1 (*ATP8B1*) which encodes the protein amino phospholipid translocase, which is vital for bile acid homeostasis in the liver. Bile acids have been suggested as potential biological contributors to AD as they associate with AD biomarkers and are increased in AD [288]. Taken together, these studies indicate that resilient donors might have a lower genetic risk for AD and possibly have specific protective SNPs, although large cohort studies are required to increase sample sizes to replicate current targets. Moreover, beyond identifying susceptibility or protective genes in AD, understanding the mechanisms through which these variants influence reserve capability will be essential to identify key processes in maintaining cognition.

#### Changes in lipids

Large-scale GWAS for AD have identified several risk loci and pathways related to lipid metabolism, such as *ApoE*, ATP-binding cassette transporter 1 *(ABCA1)* or *ABCA7*. As lipids provide scaffolds for neurotransmission and atrophy in AD involves the loss of structural lipids, they are likely to play a role in resilient individuals, in which often atrophy is absent and neuronal health preserved. Increased amounts of diacylglycerol (DAG) and monoacylglycerol (MAG) lipases were found in AD patients while the levels in resilient donors were comparable to those of control [289]. DAG pools are tightly regulated as they serve multiple critical roles including precursors for structural glycerophospholipids and MAG, mediators of signal transduction via activation of protein kinase C, protein kinase D, Ca2 + /calmodulin-dependent protein kinase II (CaMKII) RasGRP1/Ras/Erk MAPK, chimaerins, and munc13 proteins that regulate neurotransmitter release, regulation of immunological synapse function and ROS production in microglia. The differences in DAG pools between resilient and AD donors is a clear example of an upstream process involved in a plethora of metabolic processes, making it impossible to distinguish if it would be related to resilience or resistance, or CR or BM. In addition, by identifying resilient Tg2576 animals by comparing learners and non-learners, PLA2G4E has been put forward as a candidate gene for resilience [35]. Higher levels of this phospholipase were found in the hippocampus of resilient mice compared to impaired animals. Furthermore, increased PLA2G4E has also been demonstrated in the temporal cortex of cognitively intact patients with AD pathology (Braak II-IV) and in control donors compared to AD patients (Braak V-VI), while upregulation restored cognitive deficits in APP/PS1 animals and increased spine density.

#### Sex differences

Approximately two-thirds of patients diagnosed with AD are female [290], which also holds true after controlling for age [291]. Numerous studies have demonstrated that sex might play an important role in the pathophysiology of AD [292]. In animal models for AD, females display learning deficits earlier than males [293] and blocking microglial proliferation with colony-stimulating factor-1 (*CSF1R*) inhibitors only led to a reduction in tau and to gene-expression patters similar to WT animals in females, but not in males [294]. In cognitively intact individuals, women have more AD pathology than men [295] and markers related to estrogenic/androgenic and neuronal activity differed between sexes [296]. It is therefore not unlikely that resilience to AD could be attributed to different cellular and molecular mechanisms depending on sex. Recently, a female-specific SNP was found located near GATA Binding Protein 3 (GATA3), which correlated with higher resilience scores in females [76]. GATA3 is a transcription factor that has been related to estrogen receptor signaling and regulation of T cells. Differences in sex should be considered when investigating potential mechanisms related to resilience.

### Concluding remarks

It is evident that some individuals are able to withstand significant amounts of AD neuropathology while maintaining cognition. In both humans and animal models it has been well established that cognitive stimuli and lifestyle factors have a positive influence on cognition. The concept of reserve or resilience remains widely debated as is evident from the different criteria and definitions that are used. Novel methods have allowed to identify resilient subjects with more precision during life by using imaging or fluid-based biomarkers and the advancing implementation of omics will help to identify novel molecular changes that could explain resilience mechanistically. Regardless, only when a full picture of all pathological lesions and measurements of cognitive performance over time are available, distinctions can be made between true resilient individuals or those who are likely to develop dementia. This is currently only feasible in a few large-scale longitudinal cohorts.

**Fig. 3:**
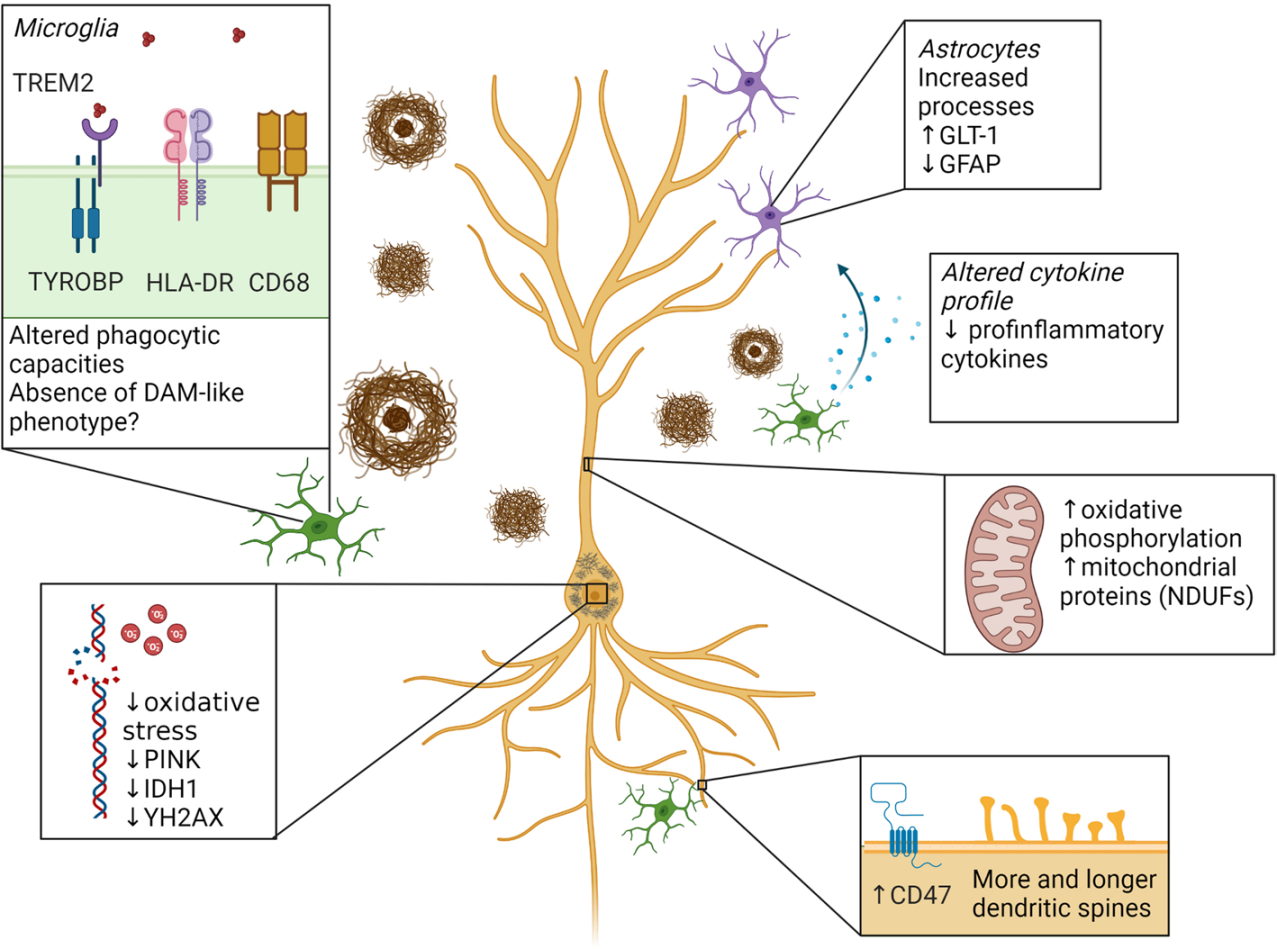
Schematic overview of most pronounced cellular and molecular changes in resilient individuals compared to AD patients. Compared to AD patients, multiple studies have indicated a different glial reaction to AD pathology in resilient donors. A reduction in the total number of microglia based on the markers TREM2, HLA-DR or CD86 have been found in resilient donors, while others have shown an increase of these markers near plaques in resilient donors, indicating a more phagocytotic subpopulation of microglia. While it has been hypothesized that microglia lack a DAM-like phenotype in resilience, this remains to be substantiated. In astrocytes, more homeostatic properties have been found, such as increased processes, increased expression of GLT-1 and reduced amounts of GFAP. Furthermore, an altered cytokine profile was observed in resilient donors. Reductions of both oxidative stress and DNA damage was observed based on the markers PINK, IDH1 and YH2AX. Mitochondria dysfunction was reduced in resilient donors, as increased oxidative phosphorylation and mitochondrial subunits were increased compared to AD patients. Increased levels of the “do not eat me signal” CD47 and increased levels and altered morphologies of dendritic spines were found in resilient donors compared to AD. Abbreviations: CD47; cluster of differentiation 47, DAM; damage associated microglia, CD68; cluster of differentiation 68, GLT-1; glutamate transporter-1, GFAP; glial fibrillary acidic protein, HLA-DR; major histocompatibility complex, class II, DR alpha, IDH1; isocitrate dehydrogenase (NADP( +)) 1, NDUFs; NADH:ubiquinone oxidoreductase core subunits, PINK; PTEN-induced kinase 1, TYROBP; TYRO protein tyrosine kinase-binding protein, YH2AX; H2A histone family member X

Fortunately, most studies focussing on molecular and cellular mechanisms related to resilience are pointing in similar directions. Differences in synaptic signalling, such as the maintenance of presynaptic SNARE proteins (Fig. 2), glial reactions to AD pathology, genetic background and changes in mitochondria are becoming increasingly more apparent to play a prominent role in mediating resilience (Fig. 3). This suggests a potential biological substrate of resilience, in which the cellular responses to AD pathology in both neurons and glial cells remain homeostatic. These responses prevent the transition into irreversible states that could otherwise contribute to the progression of the disease. Importantly, to what extent the observed changes are causal or simply correlate with resilient individuals should be further established. Interestingly, lifestyle factors seem to influence both resilience and resistance, or CR and BM, as they are both associated with maintaining brain functions despite the presence of AD pathology or with lower amounts of AD pathology than expected. It can be hypothesized that these phenomena are part of the same biological spectrum, in which individuals initially have more resistance to AD pathology until pathology can no longer be cleared and resilience comes into play. We postulate that both resistant and resilient mechanisms can help to maintain cognition until a certain tipping point, at which the levels of pathology or disease-related processes become too severe. This has also been illustrated by the fact that there are no cognitively intact centenarians with end-stage amounts of AD pathology [24]. Furthermore, it might be possible that not all resilient donors share the same mechanisms to remain cognitively intact. For example, differences in sex might play a role, or the presence of specific genetic risk factors could lead to different molecular subtypes for resilience, which was recently shown to be true for AD patients [297].

Ultimately, by elucidating the molecular and cellular mechanisms, novel drug targets can be identified. However, as most identified mechanisms are currently derived from post-mortem tissue, causality is often lacking and should be prioritized. One example of how investigating resilience individuals can help with identifying novel therapeutic approaches was the observation of the cellular senescence stress response in NFT-bearing neurons [298], which was higher in AD patients than resilient individuals [98]. This observation has led to a clinical pilot to test senolytic drugs, which clear senescent cells, in AD [299]. Finally, one important factor when attempting to activate resilient mechanism in AD patients, is disease stage. It might be possible that once the disease has progressed too far that activating resilient or resistant mechanisms is no longer successful, as it is uncertain if resilient donors themselves remain cognitively intact with end-stage amounts of AD pathology.

The question remains how lifestyle factors ultimately lead to molecular and cellular changes. Future studies should carefully characterize resilient individuals, confirm their findings in different cohorts and determine the causality of their findings. Only then processes that might be neuroprotective against AD can be discriminated. Eventually, newly identified targets that could explain resilience should ultimately lead to novel avenues of therapeutic developments of AD, by activating mechanism of resilience in AD patients.

## Data Availability

Not applicable.

## Abbreviations

AHN: Adult human neurogenesis
ApoE: Apolipoprotein E
AD: Alzheimer’s disease
AMPA: α-Amino-3-hydroxy-5-methyl-4-isoxazolepropionic acid
GluA1: AMPA Type Subunit 1
APP: Amyloid precursor protein
ATP8B1: ATPase phospholipid transporting 8B1
ABCA7: ATP binding cassette subfamily A member 7
ABCA1: ATP-binding cassette transporter 1
oAβ: Aβ oligomers
BLSA: Baltimore Longitudinal Study of Aging
BDNF: Brain derived neurotrophic factor
BR: Brain reserve
BM: Brain maintenance
Aβ: β-Amyloid
CAMKIIα: Calcium/calmodulin-dependent protein kinase type II subunit alpha
*Ctsh*: Cathepsin H
CVD: Cerebrovascular disease
CSF: Cerebrospinal fluid
CLU: Clusterin
CFAS: Cognitive Function and Ageing Study
CR: Cognitive reserve
CSF1R: Colony-stimulating factor-1
cAMP: Cyclic AMP
DAG: Diacylglycerol
DAM: Disease-associated microglia
DLPFC: Dorsolateral prefrontal cortex
DCX: Doublecortin
ENC1: Ectodermal-neural cortex 1
EE: Enriched environment
fAD: Familial AD
Fcgr2b: Fc region receptor II-b
FGF2: Fibroblast growth factor 2
FDG-PET: Fluoro-2-deoxyglucose positron-emission tomography
GATA3: GATA Binding Protein 3
GFAP: Glial fibrillary acidic protein
GRIN2A: Glutamate ionotropic receptor NMDA type subunit 2A
GLT-1: Glutamate transporter-1
GAP-43: Growth-associated protein 43
GWAS: Genome-wide association studies
Hcls1: Hematopoietic cell-specific Lyn substrate 1
HS: Hippocampal sclerosis
HAAS: Honolulu-Asia Aging Study
ptau: Hyperphosphorylated tau
Itgb2: Integrin subunit beta 2
IL: Interleukin
NADP( +)) 1 (IDH1: Isocitrate Dehydrogenase
KRT10: Keratin type 1
Ki-67: Antigen Kiel 67
LOAD: Late-onset AD
LBs: Lewy bodies
MnSOD: Manganese superoxide dismutase
MAP: Memory and Aging Project
mGluR3: Metabortopic glutamate receptor 3
miRNAs: MicroRNAs
MAPT: Microtubule-associated protein tau
MFN2: Mitofusin 2
MAG: Monoacylglycerol
MEF2: Myocyte enhancer factor-2
NDAN: Non-demented Alzheimer neuropathology
NGF: Nerve growth factor
VGF: Nerve growth factor inducible
NRN1: Neuritin
NPs: Neuritic plaques
NFL-L: Neurofilament light chain
NFTs: Neurofibrillary tangles
NLRP3: NOD-, LRR- and pyrin domain-containing 3
PICALM: Phosphatidylinositol binding clathrin assembly protein
PLA2G4E: Phospholipase A2 Group IVE
PLGC2: Phospholipase C gamma 2
pCREB: Phosphorylated cAMP response element binding protein
pTDP-43: Phosphorylated TAR DNA-binding protein 43
PEDF: Pigment epithelium-derived factor
PiB: Pittsburgh Compound-B
PDGF: Platelet-derived growth factor
PIGs: Plaque-induced genes
PET: Positron emission tomography
PSD-95: Postsynaptic density 95
PS1: Presenilin 1
PS2: Presenilin 2
PCNA: Proliferating cell nuclear antigen
PINK1: PTEN induced kinase 1
RORB: RAR Related Orphan Receptor B
ROS: Religious Orders Study
RNASE13: Ribonuclease A family member 13
RPH3A: Rabphilin 3A
SNARE: Soluble N-ethylmaleimide-sensitive-factor attachment protein receptor
SNPs: Single-nucleotide polymorphism
SLC17A6: Solute carrier family 17 member 6
SAP-97: Synapse associated protein 97
SYP: Synaptophysin
SNAP25: Synaptosome-associated protein of 25 kDa
SYN1: Synapsin-1
STX1A/B: Syntaxin-1A and 1B
SYT12: Synaptotagmin 12
STXBP1: Syntaxin binding protein 1
SOX2: SRY-Box Transcription Factor 2
TMEM106B: Transmembrane protein 106B
TREM2: Triggering receptor expressed on myeloid cells 2
TNF-α: Tumor necrosis factor-α
TYROBP: Tyrosine kinase binding protein
UBA1: Ubiquitin-like modifier activating enzyme 1
UNC5C: Unc-5 netrin receptor C
VEGFa: Vascular endothelial growth factor A
VAT1: Vesicle amine transport 1
VAMP1/2: Vesicle-associated membrane proteins 1/2
VAMP5: Vesicle associated membrane protein 5
ZnT3: Zinc transporter 3

## References

[1] Vandenberghe R (2014). The relationship between amyloid deposition, neurodegeneration, and cognitive decline in dementia. Curr Neurol Neurosci Rep.

[2] Terracciano A (2014). Personality and risk of Alzheimer’s disease: new data and meta-analysis. Alzheimer’s Dement.

[3] Baumgart M (2015). Summary of the evidence on modifiable risk factors for cognitive decline and dementia: a population-based perspective. Alzheimer’s Dement.

[4] Xu W (2015). Meta-analysis of modifiable risk factors for Alzheimer’s disease. J Neurol Neurosurg Psychiatry.

[5] Swaab DF (1991). Brain aging and Alzheimer’s disease, “wear and tear” versus “use it or lose it”. Neurobiol Aging.

[6] Xiang C, Zhang Y. Comparison of cognitive intervention strategies for individuals with Alzheimer’s disease: a systematic review and network meta-analysis. Neuropsychol Rev. 2023. 10.1007/s11065-023-09584-5 10.1007/s11065-023-09584-5 PMC11166762 36929474

[7] Barnes DE, Yaffe K (2011). The projected effect of risk factor reduction on Alzheimer’s disease prevalence. Lancet Neurol.

[8] Zhu Q-B, Bao A-M, Swaab D (2019). Activation of the brain to postpone dementia: a concept originating from postmortem human brain studies. Neurosci Bull.

[9] Azevedo CV (2023). The effects of resistance exercise on cognitive function, amyloidogenesis, and neuroinflammation in Alzheimer’s disease. Front Neurosci.

[10] Wang M, Zhang H, Liang J, Huang J, Chen N (2023). Exercise suppresses neuroinflammation for alleviating Alzheimer’s disease. J Neuroinflammation.

[11] Nelson PT (2012). Correlation of Alzheimer disease neuropathologic changes with cognitive status: a review of the literature. J Neuropathol Exp Neurol.

[12] Blessed G, Tomlinson BE, Roth M (1968). The association between quantitative measures of dementia and of senile change in the cerebral grey matter of elderly subjects. Br J psychiatry.

[13] Katzman R (1988). Clinical, pathological, and neurochemical changes in dementia: a subgroup with preserved mental status and numerous neocortical plaques. Ann Neurol Off J Am Neurol Assoc Child Neurol Soc.

[14] Price JL, Morris JC (1999). Tangles and plaques in nondemented aging and “preclinical” Alzheimer’s disease. Ann Neurol Off J Am Neurol Assoc Child Neurol Soc.

[15] Riley KP, Snowdon DA, Markesbery WR (2002). Alzheimer’s neurofibrillary pathology and the spectrum of cognitive function: findings from the Nun study. Ann Neurol.

[16] Ahangari N, Fischer CE, Schweizer TA, Munoz DG (2023). Cognitive resilience and severe Alzheimer’s disease neuropathology. Aging Brain.

[17] Cfas MRC (2001). Pathological correlates of late-onset dementia in a multicentre, community-based population in England and Wales. Lancet.

[18] Zhang M (1990). The prevalence of dementia and Alzheimer’s disease in Shanghai, China: impact of age, gender, and education. Ann Neurol Off J Am Neurol Assoc Child Neurol Soc.

[19] Stern Y (1994). Influence of education and occupation on the incidence of Alzheimer’s disease. JAMA.

[20] McGurn B, Deary IJ, Starr JM (2008). Childhood cognitive ability and risk of late-onset Alzheimer and vascular dementia. Neurology.

[21] Scarmeas N, Levy G, Tang M-X, Manly J, Stern Y (2001). Influence of leisure activity on the incidence of Alzheimer’s disease. Neurology.

[22] Arenaza-Urquijo EM, Vemuri P (2018). Resistance vs resilience to Alzheimer disease: clarifying terminology for preclinical studies. Neurology.

[23] Ganz AB (2018). Neuropathology and cognitive performance in self-reported cognitively healthy centenarians. Acta Neuropathol Commun.

[24] Zhang M et al*. *Resilience and resistance to the accumulation of amyloid plaques and neurofibrillary tangles in centenarians: An age-continuous perspective. Alzheimer’s Dement. 2022. 10.1002/alz.12899. 10.1002/alz.12899 36583547

[25] Stern Y (2023). A framework for concepts of reserve and resilience in aging. Neurobiol Aging.

[26] Nyberg L, Lövdén M, Riklund K, Lindenberger U, Bäckman L (2012). Memory aging and brain maintenance. Trends Cogn Sci.

[27] Tang X, Varma VR, Miller MI, Carlson MC (2017). Education is associated with sub-regions of the hippocampus and the amygdala vulnerable to neuropathologies of Alzheimer’s disease. Brain Struct Funct.

[28] Arenaza-Urquijo EM (2013). Relationships between years of education and gray matter volume, metabolism and functional connectivity in healthy elders. Neuroimage.

[29] Silles MA (2009). The causal effect of education on health: evidence from the United Kingdom. Econ Educ Rev.

[30] Alty JE (2023). Sex-specific protective effects of cognitive reserve on age-related cognitive decline. Neurology.

[31] Ersoezlue E (2023). Lifelong experiences as a proxy of cognitive reserve moderate the association between connectivity and cognition in Alzheimer’s disease. Neurobiol Aging.

[32] Soldan A (2017). Cognitive reserve and long-term change in cognition in aging and preclinical Alzheimer’s disease. Neurobiol Aging.

[33] Soldan A (2020). Cognitive reserve and midlife vascular risk: Cognitive and clinical outcomes. Ann Clin Transl Neurol.

[34] Montaron M, Charrier V, Blin N, Garcia P, Abrous DN (2020). Responsiveness of dentate neurons generated throughout adult life is associated with resilience to cognitive aging. Aging Cell.

[35] Pérez-González M (2020). PLA2G4E, a candidate gene for resilience in Alzheimer´s disease and a new target for dementia treatment. Prog Neurobiol.

[36] Martinez-Coria H (2015). Repeated cognitive stimulation alleviates memory impairments in an Alzheimer’s disease mouse model. Brain Res Bull.

[37] Lonnemann N, Korte M, Hosseini S (2023). Repeated performance of spatial memory tasks ameliorates cognitive decline in APP/PS1 mice. Behav Brain Res.

[38] Sierksma ASR (2013). Behavioral and neurobiological effects of prenatal stress exposure in male and female APPswe/PS1dE9 mice. Neurobiol Aging.

[39] Selvi Y (2017). Impact of enriched environment on production of tau, amyloid precursor protein and amyloid-β peptide in high-fat and high-sucrose-fed rats. Acta Neuropsychiatr.

[40] Cattaud V (2018). Early disruption of parvalbumin expression and perineuronal nets in the hippocampus of the Tg2576 mouse model of Alzheimer’s disease can be rescued by enriched environment. Neurobiol Aging.

[41] Lesuis SL, Weggen S, Baches S, Lucassen PJ, Krugers HJ (2018). Targeting glucocorticoid receptors prevents the effects of early life stress on amyloid pathology and cognitive performance in APP/PS1 mice. Transl Psychiatry.

[42] Yegla B, Foster TC (2022). Operationally defining cognitive reserve genes. Neurobiol Aging.

[43] Heneka MT (2015). Neuroinflammation in Alzheimer’s disease. Lancet Neurol.

[44] De Strooper B, Karran E (2016). The cellular phase of Alzheimer’s disease. Cell.

[45] Otero-Garcia M (2022). Molecular signatures underlying neurofibrillary tangle susceptibility in Alzheimer’s disease. Neuron.

[46] Leng K (2021). Molecular characterization of selectively vulnerable neurons in Alzheimer’s disease. Nat Neurosci.

[47] Vijayaragavan K (2022). Single-cell spatial proteomic imaging for human neuropathology. Acta Neuropathol Commun.

[48] Montine TJ (2019). Concepts for brain aging: resistance, resilience, reserve, and compensation. Alzheimers Res Ther.

[49] Flor-García M (2020). Unraveling human adult hippocampal neurogenesis. Nat Protoc.

[50] Braak H, Braak EVA (1995). Staging of Alzheimer’s disease-related neurofibrillary changes. Neurobiol Aging.

[51] Mirra SS (1991). The consortium to establish a registry for Alzheimer’s Disease (CERAD): Part II. Standardization of the neuropathologic assessment of Alzheimer’s disease. Neurology.

[52] Azarpazhooh MR (2019). A third of community-dwelling elderly with intermediate and high level of Alzheimer’s neuropathologic changes are not demented: A meta-analysis. Ageing Res Rev.

[53] Taddei RN (2022). Changes in glial cell phenotypes precede overt neurofibrillary tangle formation, correlate with markers of cortical cell damage, and predict cognitive status of individuals at Braak III-IV stages. Acta Neuropathol Commun.

[54] Tosun D (2022). Contribution of Alzheimer’s biomarkers and risk factors to cognitive impairment and decline across the Alzheimer’s disease continuum. Alzheimer’s Dement.

[55] Grońska-Pęski M, Gonçalves JT, Hébert JM (2021). Enriched environment promotes adult Hippocampal neurogenesis through FGFRs. J Neurosci.

[56] Gerenu G, Dobarro M, Ramirez MJ, Gil-Bea FJ (2013). Early cognitive stimulation compensates for memory and pathological changes in Tg2576 mice. Biochim Biophys Acta - Mol Basis Dis.

[57] Beauquis J (2013). Environmental enrichment prevents astroglial pathological changes in the hippocampus of APP transgenic mice, model of Alzheimer’s disease. Exp Neurol.

[58] Birch AM, Kelly ÁM (2019). Lifelong environmental enrichment in the absence of exercise protects the brain from age-related cognitive decline. Neuropharmacology.

[59] Peirce JL, Lu L, Gu J, Silver LM, Williams RW (2004). A new set of BXD recombinant inbred lines from advanced intercross populations in mice. BMC Genet.

[60] Heuer SE (2020). Identifying the molecular systems that influence cognitive resilience to Alzheimer’s disease in genetically diverse mice. Learn Mem.

[61] Mostafavi S (2018). A molecular network of the aging human brain provides insights into the pathology and cognitive decline of Alzheimer’s disease. Nat Neurosci.

[62] Reinders NR (2016). Amyloid-β effects on synapses and memory require AMPA receptor subunit GluA3. Proc Natl Acad Sci.

[63] Holstege H (2018). The 100-plus Study of cognitively healthy centenarians: rationale, design and cohort description. Eur J Epidemiol.

[64] Arenaza-Urquijo EM (2019). The metabolic brain signature of cognitive resilience in the 80+: beyond Alzheimer pathologies. Brain.

[65] Arnold SE (2013). Cellular, synaptic, and biochemical features of resilient cognition in Alzheimer’s disease. Neurobiol Aging.

[66] Barker SJ (2021). MEF2 is a key regulator of cognitive potential and confers resilience to neurodegeneration. Sci Transl Med.

[67] Barroeta-Espar I (2019). Distinct cytokine profiles in human brains resilient to Alzheimer’s pathology. Neurobiol Dis.

[68] Bilousova T (2016). Synaptic amyloid-β oligomers precede p-Tau and differentiate high pathology control cases. Am J Pathol.

[69] Bjorklund NL (2012). Absence of amyloid β oligomers at the postsynapse and regulated synaptic Zn 2+ in cognitively intact aged individuals with Alzheimer’s disease neuropathology. Mol Neurodegener.

[70] Boros BD (2017). Dendritic spines provide cognitive resilience against Alzheimer’s disease. Ann Neurol.

[71] Briley D (2016). Preserved neurogenesis in non-demented individuals with AD neuropathology. Sci Rep.

[72] Buciuc M (2020). Association between TDP-43 type and cognitive resilience to Alzheimer’s disease: a case-control study. Neurobiol Aging.

[73] Cain A (2023). Multicellular communities are perturbed in the aging human brain and Alzheimer’s disease. Nat Neurosci.

[74] Carlyle BC (2021). Synaptic proteins associated with cognitive performance and neuropathology in older humans revealed by multiplexed fractionated proteomics. Neurobiol Aging.

[75] Dumitrescu L (2020). Genetic variants and functional pathways associated with resilience to Alzheimer’s disease. Brain.

[76] Eissman JM (2022). Sex differences in the genetic architecture of cognitive resilience to Alzheimer’s disease. Brain.

[77] Esparza TJ (2013). Amyloid-beta oligomerization in Alzheimer dementia versus high-pathology controls. Ann Neurol.

[78] Fracassi A (2023). TREM2 -induced activation of microglia contributes to synaptic integrity in cognitively intact aged individuals with Alzheimer’s neuropathology. Brain Pathol.

[79] Hurst C (2023). Integrated proteomics to understand the role of Neuritin (NRN1) as a mediator of cognitive resilience to Alzheimer’s disease. Mol Cell Proteomics.

[80] Iacono D (2008). Neuronal hypertrophy in asymptomatic Alzheimer disease. J Neuropathol Exp Neurol.

[81] Jiang H, Esparza TJ, Kummer TT, Brody DL (2021). Unbiased high-content screening reveals Aβ- and tau-independent synaptotoxic activities in human brain homogenates from Alzheimer’s patients and high-pathology controls. Plos One.

[82] Johnson ECB (2020). Large-scale proteomic analysis of Alzheimer’s disease brain and cerebrospinal fluid reveals early changes in energy metabolism associated with microglia and astrocyte activation. Nat Med.

[83] Kobayashi E (2018). Activated forms of astrocytes with higher GLT-1 expression are associated with cognitive normal subjects with Alzheimer pathology in human brain. Sci Rep.

[84] Latimer CS (2019). Resistance and resilience to Alzheimer’s disease pathology are associated with reduced cortical pTau and absence of limbic-predominant age-related TDP-43 encephalopathy in a community-based cohort. Acta Neuropathol Commun.

[85] Lee DH (2019). Neural substrates of cognitive reserve in Alzheimer’s disease spectrum and normal aging. Neuroimage.

[86] Lee CS (2021). Application of deep learning to understand resilience to Alzheimer’s disease pathology. Brain Pathol..

[87] Maarouf CL (2011). Alzheimer’s disease and non-demented high pathology control nonagenarians: comparing and contrasting the biochemistry of cognitively successful aging. Plos One.

[88] Mathys H (2023). Single-cell atlas reveals correlates of high cognitive function, dementia, and resilience to Alzheimer’s disease pathology. Cell.

[89] Neuner SM, Wilmott LA, Hoffmann BR, Mozhui K, Kaczorowski CC (2017). Hippocampal proteomics defines pathways associated with memory decline and resilience in normal aging and Alzheimer’s disease mouse models. Behav Brain Res.

[90] Montine TJ (2022). Association of cognition and dementia with neuropathologic changes of Alzheimer disease and other conditions in the oldest old. Neurology.

[91] Perez-Nievas BG (2013). Dissecting phenotypic traits linked to human resilience to Alzheimer’s pathology. Brain.

[92] Ramos-Miguel A (2021). Proteomic identification of select protein variants of the SNARE interactome associated with cognitive reserve in a large community sample. Acta Neuropathol.

[93] Scheff SW, Ansari MA, Mufson EJ (2016). Oxidative stress and hippocampal synaptic protein levels in elderly cognitively intact individuals with Alzheimer’s disease pathology. Neurobiol Aging.

[94] Seyfried NT (2017). A multi-network approach identifies protein-specific co-expression in asymptomatic and symptomatic Alzheimer’s disease. Cell Syst.

[95] Silva ART (2014). Repair of oxidative DNA damage, cell-cycle regulation and neuronal death may influence the clinical manifestation of Alzheimer’s disease. Plos One.

[96] Singh A (2020). Functional integrity of synapses in the central nervous system of cognitively intact individuals with high Alzheimer’s disease neuropathology is associated with absence of Synaptic Tau Oligomers. J Alzheimer’s Dis.

[97] Taddei RN (2023). Tau Oligomer-containing Synapse elimination by microglia and astrocytes in Alzheimer disease. JAMA Neurol.

[98] Walker JM (2022). Differential protein expression in the hippocampi of resilient individuals identified by digital spatial profiling. Acta Neuropathol Commun.

[99] Yu L (2020). Cortical proteins associated with cognitive resilience in community-dwelling older persons. JAMA Psychiat.

[100] Zolochevska O, Bjorklund N, Woltjer R, Wiktorowicz JE, Taglialatela G (2018). Postsynaptic proteome of non-demented individuals with Alzheimer’s disease neuropathology. J Alzheimer’s Dis.

[101] Zammit AR (2022). Cortical proteins and individual differences in cognitive resilience in older adults. Neurology.

[102] O’Brien RJ (2009). Neuropathologic studies of the Baltimore longitudinal study of aging (BLSA). J Alzheimer’s Dis.

[103] Iacono D (2009). The Nun study: clinically silent AD, neuronal hypertrophy, and linguistic skills in early life. Neurology.

[104] Bennett DA (2018). Religious orders study and rush memory and aging project. J Alzheimer’s Dis.

[105] Head E (2009). Synaptic proteins, neuropathology and cognitive status in the oldest-old. Neurobiol Aging.

[106] White L (2009). Brain lesions at autopsy in older Japanese-American men as related to cognitive impairment and dementia in the final years of life: a summary report from the Honolulu-Asia aging study. J Alzheimer’s Dis.

[107] Savva GM (2009). Age, neuropathology, and dementia. N Engl J Med.

[108] Sepulveda-Falla D (2023). Resistant and resilient mutations in protection against familial Alzheimer’s disease: learning from nature. Mol Neurodegener.

[109] Sepulveda-Falla D (2022). Distinct tau neuropathology and cellular profiles of an APOE3 Christchurch homozygote protected against autosomal dominant Alzheimer’s dementia. Acta Neuropathol.

[110] Lopera F (2023). Resilience to autosomal dominant Alzheimer’s disease in a Reelin-COLBOS heterozygous man. Nat Med.

[111] Jack CR (2018). NIA-AA research framework: toward a biological definition of Alzheimer’s disease. Alzheimer’s Dement.

[112] Aizenstein HJ (2008). Frequent amyloid deposition without significant cognitive impairment among the elderly. Arch Neurol.

[113] Rowe CC (2010). Amyloid imaging results from the Australian Imaging, Biomarkers and Lifestyle (AIBL) study of aging. Neurobiol Aging.

[114] Lister-James, J. *et al.* Florbetapir f-18: a histopathologically validated Beta-amyloid positron emission tomography imaging agent. in *Seminars in nuclear medicine* vol. 41 300–304 (Elsevier, 2011). 10.1053/j.semnuclmed.2011.03.001 21624563

[115] De Meyer G (2010). Diagnosis-independent Alzheimer disease biomarker signature in cognitively normal elderly people. Arch Neurol.

[116] Thal DR, Capetillo-Zarate E, Del Tredici K, Braak H (2006). The development of amyloid beta protein deposits in the aged brain. Sci aging Knowl Environ.

[117] Serrano-Pozo A, Frosch MP, Masliah E, Hyman BT (2011). Neuropathological alterations in Alzheimer disease. Cold Spring Harb Perspect Med.

[118] Duyckaerts C, Delatour B, Potier M-C (2009). Classification and basic pathology of Alzheimer disease. Acta Neuropathol.

[119] Knowles RB (1999). Plaque-induced neurite abnormalities: implications for disruption of neural networks in Alzheimer’s disease. Proc Natl Acad Sci.

[120] Malek-Ahmadi M, Perez SE, Chen K, Mufson EJ (2016). Neuritic and diffuse plaque associations with memory in non-cognitively impaired elderly. J Alzheimer’s Dis.

[121] Henkins KM (2012). Extensive p-tau pathology and SDS-stable p-tau oligomers in Alzheimer’s cortical synapses. Brain Pathol.

[122] Selkoe, D. J. Soluble oligomers of the amyloid β-protein: Impair synaptic plasticity and behavior. in *Synaptic Plasticity and the Mechanism of Alzheimer’s Disease* 89–102 (Springer, 2008). 10.1016/j.bbr.2008.02.016 PMC2601528 18359102

[123] Sokolow S (2012). AD synapses contain abundant Aβ monomer and multiple soluble oligomers, including a 56-kDa assembly. Neurobiol Aging.

[124] Kapasi A, DeCarli C, Schneider JA (2017). Impact of multiple pathologies on the threshold for clinically overt dementia. Acta Neuropathol.

[125] Spires-Jones TL, Attems J, Thal DR (2017). Interactions of pathological proteins in neurodegenerative diseases. Acta Neuropathol.

[126] Kawas CH (2015). Multiple pathologies are common and related to dementia in the oldest-old: The 90+ Study. Neurology.

[127] Bennett DA, Wilson RS, Boyle PA, Buchman AS, Schneider JA (2012). Relation of neuropathology to cognition in persons without cognitive impairment. Ann Neurol.

[128] Boyle PA (2013). Much of late life cognitive decline is not due to common neurodegenerative pathologies. Ann Neurol.

[129] Yu L (2015). Residual decline in cognition after adjustment for common neuropathologic conditions. Neuropsychology.

[130] Xu H (2019). Association of lifespan cognitive reserve indicator with dementia risk in the presence of brain pathologies. JAMA Neurol.

[131] Robinson JL (2018). Non-Alzheimer’s contributions to dementia and cognitive resilience in The 90+ Study. Acta Neuropathol.

[132] Rentz DM (2010). Cognition, reserve, and amyloid deposition in normal aging. Ann Neurol.

[133] Vemuri P (2012). Effect of lifestyle activities on Alzheimer disease biomarkers and cognition. Ann Neurol.

[134] Landau SM (2012). Association of lifetime cognitive engagement and low β-amyloid deposition. Arch Neurol.

[135] Estanga A (2017). Beneficial effect of bilingualism on Alzheimer’s disease CSF biomarkers and cognition. Neurobiol Aging.

[136] Lo RY, Jagust WJ (2013). Effect of cognitive reserve markers on Alzheimer pathological progression. Alzheimer Dis Assoc Disord.

[137] Brayne C (2010). Education, the brain and dementia: neuroprotection or compensation?. Brain.

[138] Wilson RS, Scherr PA, Schneider JA, Tang Y, Bennett DA (2007). Relation of cognitive activity to risk of developing Alzheimer disease. Neurology.

[139] Aiello Bowles EJ (2019). Cognitive resilience to Alzheimer’s disease pathology in the human brain. J Alzheimer’s Dis.

[140] Wilson RS (2019). Education and cognitive reserve in old age. Neurology.

[141] Pettigrew C (2020). Cognitive reserve and rate of change in Alzheimer’s and cerebrovascular disease biomarkers among cognitively normal individuals. Neurobiol Aging.

[142] Lazarov O (2005). Environmental enrichment reduces Aβ levels and amyloid deposition in transgenic mice. Cell.

[143] Cracchiolo JR (2007). Enhanced cognitive activity—over and above social or physical activity—is required to protect Alzheimer’s mice against cognitive impairment, reduce Aβ deposition, and increase synaptic immunoreactivity. Neurobiol Learn Mem.

[144] Arendash GW (2004). Environmental enrichment improves cognition in aged Alzheimer’s transgenic mice despite stable β-amyloid deposition. NeuroReport.

[145] Jeong YH (2011). Environmental enrichment compensates for the effects of stress on disease progression in Tg2576 mice, an Alzheimer’s disease model. J Neurochem.

[146] Jack CR (2000). Rates of hippocampal atrophy correlate with change in clinical status in aging and AD. Neurology.

[147] Uylings HBM, De Brabander JM (2002). Neuronal changes in normal human aging and Alzheimer’s disease. Brain Cogn.

[148] Raz N (2005). Regional brain changes in aging healthy adults: general trends, individual differences and modifiers. Cereb cortex.

[149] Desikan RS (2010). Selective disruption of the cerebral neocortex in Alzheimer’s disease. Plos One.

[150] Piras F, Cherubini A, Caltagirone C, Spalletta G (2011). Education mediates microstructural changes in bilateral hippocampus. Hum Brain Mapp.

[151] Staff RT (2012). Childhood socioeconomic status and adult brain size: childhood socioeconomic status influences adult hippocampal size. Ann Neurol.

[152] Gidicsin CM (2015). Cognitive activity relates to cognitive performance but not to Alzheimer disease biomarkers. Neurology.

[153] Foubert-Samier A (2012). Education, occupation, leisure activities, and brain reserve: a population-based study. Neurobiol Aging.

[154] Markham JA, Greenough WT (2004). Experience-driven brain plasticity: Beyond the synapse. Neuron Glia Biol.

[155] Chetelat G (2010). Larger temporal volume in elderly with high versus low beta-amyloid deposition. Brain.

[156] Wirth M (2013). The effect of amyloid β on cognitive decline is modulated by neural integrity in cognitively normal elderly. Alzheimer’s Dement.

[157] Boots EA (2015). Occupational complexity and cognitive reserve in a middle-aged cohort at risk for Alzheimer’s disease. Arch Clin Neuropsychol.

[158] Dickerson BC (2009). The cortical signature of Alzheimer’s disease: regionally specific cortical thinning relates to symptom severity in very mild to mild AD dementia and is detectable in asymptomatic amyloid-positive individuals. Cereb cortex.

[159] Arenaza-Urquijo EM (2013). Cognitive reserve proxies relate to gray matter loss in cognitively healthy elderly with abnormal cerebrospinal fluid amyloid-β levels. J Alzheimer’s Dis.

[160] Valenzuela MJ (2012). Multiple biological pathways link cognitive lifestyle to protection from dementia. Biol Psychiatry.

[161] Boros BD, Greathouse KM, Gearing M, Herskowitz JH (2019). Dendritic spine remodeling accompanies Alzheimer’s disease pathology and genetic susceptibility in cognitively normal aging. Neurobiol Aging.

[162] Negash S (2013). Resilient brain aging: characterization of discordance between Alzheimer’s disease pathology and cognition. Curr Alzheimer Res.

[163] Ziv NE, Smith SJ (1996). Evidence for a role of dendritic filopodia in synaptogenesis and spine formation. Neuron.

[164] Jung CKE, Herms J (2014). Structural dynamics of dendritic spines are influenced by an environmental enrichment: an in vivo imaging study. Cereb Cortex.

[165] Leuner B, Falduto J, Shors TJ (2003). Associative memory formation increases the observation of dendritic spines in the Hippocampus. J Neurosci.

[166] Dubelaar EJG (2006). Increased metabolic activity in nucleus basalis of Meynert neurons in elderly individuals with mild cognitive impairment as indicated by the size of the Golgi apparatus. J Neuropathol Exp Neurol.

[167] Bossers K (2010). Concerted changes in transcripts in the prefrontal cortex precede neuropathology in Alzheimer’s disease. Brain.

[168] Franzmeier N, Duering M, Weiner M, Dichgans M, Ewers M (2017). Left frontal cortex connectivity underlies cognitive reserve in prodromal Alzheimer disease. Neurology.

[169] Franzmeier N (2018). The left frontal cortex supports reserve in aging by enhancing functional network efficiency. Alzheimers Res Ther.

[170] Liang X (2020). Functional connectivity of Hippocampal CA3 predicts neurocognitive aging via CA1–frontal circuit. Cereb Cortex.

[171] Hijazi S (2020). Early restoration of parvalbumin interneuron activity prevents memory loss and network hyperexcitability in a mouse model of Alzheimer’s disease. Mol Psychiatry.

[172] Verret L (2012). Inhibitory interneuron deficit links altered network activity and cognitive dysfunction in Alzheimer model. Cell.

[173] Riudavets MA (2007). Resistance to Alzheimer’s pathology is associated with nuclear hypertrophy in neurons. Neurobiol Aging.

[174] Yu N-N, Tan M-S, Yu J-T, Xie A-M, Tan L (2016). The role of Reelin signaling in Alzheimer’s disease. Mol Neurobiol.

[175] Botella-López A (2010). β-amyloid controls altered Reelin expression and processing in Alzheimer’s disease. Neurobiol Dis.

[176] Kelley CM, Ginsberg SD, Liang WS, Counts SE, Mufson EJ (2022). Posterior cingulate cortex reveals an expression profile of resilience in cognitively intact elders. Brain Commun.

[177] Lisman J, Schulman H, Cline H (2002). The molecular basis of CaMKII function in synaptic and behavioural memory. Nat Rev Neurosci.

[178] Kessels HW, Malinow R (2009). Synaptic AMPA receptor plasticity and behavior. Neuron.

[179] Makino H, Malinow R (2011). Compartmentalized versus global synaptic plasticity on dendrites controlled by experience. Neuron.

[180] Neuner SM, Heuer SE, Zhang J-G, Philip VM, Kaczorowski CC (2019). Identification of pre-symptomatic gene signatures that predict resilience to cognitive decline in the genetically diverse AD-BXD model. Front Genet.

[181] Ren Q-G (2019). Spatial training ameliorates long-term Alzheimer’s disease-like pathological deficits by reducing NLRP3 inflammasomes in PR5 Mice. Neurotherapeutics.

[182] Stuart KE (2017). Mid-life environmental enrichment increases synaptic density in CA1 in a mouse model of Aβ-associated pathology and positively influences synaptic and cognitive health in healthy ageing. J Comp Neurol.

[183] Wei Z (2020). Environmental enrichment prevents Aβ oligomer-induced synaptic dysfunction through mirna-132 and hdac3 signaling pathways. Neurobiol Dis.

[184] Dore K (2021). PSD-95 protects synapses from β-amyloid. Cell Rep.

[185] Honer WG (2012). Cognitive reserve, presynaptic proteins and dementia in the elderly. Transl Psychiatry.

[186] Ramos-Miguel A (2017). Presynaptic proteins complexin-I and complexin-II differentially influence cognitive function in early and late stages of Alzheimer’s disease. Acta Neuropathol.

[187] Liang WS (2010). Neuronal gene expression in non-demented individuals with intermediate Alzheimer’s disease neuropathology. Neurobiol Aging.

[188] Lim KH, Kim YK, Chang Y-T (2007). Investigations of the Molecular Mechanism of Metal-Induced Aβ (1–40) Amyloidogenesis. Biochemistry.

[189] Noy D (2008). Zinc-amyloid β interactions on a millisecond time-scale stabilize non-fibrillar Alzheimer-related species. J Am Chem Soc.

[190] Deshpande A, Kawai H, Metherate R, Glabe CG, Busciglio J (2009). A role for synaptic zinc in activity-dependent Aβ oligomer formation and accumulation at excitatory synapses. J Neurosci.

[191] Lee Y-S, Silva AJ (2009). The molecular and cellular biology of enhanced cognition. Nat Rev Neurosci.

[192] Cline EN, Bicca MA, Viola KL, Klein WL (2018). The amyloid-β oligomer hypothesis: beginning of the third decade. J Alzheimer’s Dis.

[193] Bergmann O, Spalding KL, Frisén J (2015). Adult neurogenesis in humans. Cold Spring Harb Perspect Biol.

[194] Tosoni G (2023). Mapping human adult hippocampal neurogenesis with single-cell transcriptomics: reconciling controversy or fueling the debate?. Neuron.

[195] Kempermann G (2018). Human adult neurogenesis: evidence and remaining questions. Cell Stem Cell.

[196] Gatt A, Lee H, Williams G, Thuret S, Ballard C (2019). Expression of neurogenic markers in Alzheimer’s disease: a systematic review and metatranscriptional analysis. Neurobiol Aging.

[197] Lucassen PJ (2020). Limits to human neurogenesis—really?. Mol Psychiatry.

[198] Moreno-Jiménez EP (2019). Adult hippocampal neurogenesis is abundant in neurologically healthy subjects and drops sharply in patients with Alzheimer’s disease. Nat Med.

[199] Tobin MK (2019). Human hippocampal neurogenesis persists in aged adults and Alzheimer’s disease patients. Cell Stem Cell.

[200] Zhou Y (2022). Molecular landscapes of human hippocampal immature neurons across lifespan. Nature.

[201] Zolochevska O, Taglialatela G (2020). Selected microRNAs increase synaptic resilience to the damaging binding of the Alzheimer’s disease Amyloid Beta Oligomers. Mol Neurobiol.

[202] Micci M-A (2019). Hippocampal stem cells promotes synaptic resistance to the dysfunctional impact of amyloid beta oligomers via secreted exosomes. Mol Neurodegener.

[203] Konecna A, Heraud JE, Schoderboeck L, Raposo AASF, Kiebler MA (2009). What are the roles of microRNAs at the mammalian synapse?. Neurosci Lett.

[204] Properzi F, Ferroni E, Poleggi A, Vinci R (2015). The regulation of exosome function in the CNS: implications for neurodegeneration. Swiss Med Wkly.

[205] Asai H (2015). Depletion of microglia and inhibition of exosome synthesis halt tau propagation. Nat Neurosci.

[206] Takeda S (2015). Neuronal uptake and propagation of a rare phosphorylated high-molecular-weight tau derived from Alzheimer’s disease brain. Nat Commun.

[207] Weinstein G (2014). Serum brain-derived neurotrophic factor and the risk for dementia: the Framingham Heart Study. JAMA Neurol.

[208] Buchman AS (2016). Higher brain BDNF gene expression is associated with slower cognitive decline in older adults. Neurology.

[209] Rossi C (2006). Brain-derived neurotrophic factor (BDNF) is required for the enhancement of hippocampal neurogenesis following environmental enrichment. Eur J Neurosci.

[210] Bramham CR, Messaoudi E (2005). BDNF function in adult synaptic plasticity: the synaptic consolidation hypothesis. Prog Neurobiol.

[211] Horch HW, Katz LC (2002). BDNF release from single cells elicits local dendritic growth in nearby neurons. Nat Neurosci.

[212] Nagahara AH, Tuszynski MH (2011). Potential therapeutic uses of BDNF in neurological and psychiatric disorders. Nat Rev Drug Discov.

[213] Hsiao Y-H, Hung H-C, Chen S-H, Gean P-W (2014). Social interaction rescues memory deficit in an animal model of Alzheimer’s disease by increasing BDNF-dependent hippocampal neurogenesis. J Neurosci.

[214] Damirchi A, Hosseini F, Babaei P (2018). Mental training enhances cognitive function and BDNF more than either physical or combined training in elderly women with MCI: a small-scale study. Am J Alzheimer’s Dis Other Dementias®.

[215] Torasdotter M, Metsis M, Henriksson BG, Winblad B, Mohammed AH (1998). Environmental enrichment results in higher levels of nerve growth factor mRNA in the rat visual cortex and hippocampus. Behav Brain Res.

[216] Gelfo F (2011). Enriched Environment Improves Motor Function and Increases Neurotrophins in Hemicerebellar Lesioned Rats. Neurorehabil Neural Repair.

[217] Dandi Ε (2018). Beneficial effects of environmental enrichment on behavior, stress reactivity and synaptophysin/BDNF expression in hippocampus following early life stress. Int J Dev Neurosci.

[218] Jin K (2004). Enhanced neurogenesis in Alzheimer’s disease transgenic (PDGF-APPSw, Ind) mice. Proc Natl Acad Sci.

[219] Yabe T, Sanagi T, Yamada H (2010). The neuroprotective role of PEDF: implication for the therapy of neurological disorders. Curr Mol Med.

[220] Woodbury ME, Ikezu T (2014). Fibroblast growth factor-2 signaling in neurogenesis and neurodegeneration. J Neuroimmune Pharmacol.

[221] Shim JW, Madsen JR (2018). VEGF signaling in neurological disorders. Int J Mol Sci.

[222] Sil S, Periyasamy P, Thangaraj A, Chivero ET, Buch S (2018). PDGF/PDGFR axis in the neural systems. Mol Aspects Med.

[223] Kiyota T, Ingraham KL, Jacobsen MT, Xiong H, Ikezu T (2011). FGF2 gene transfer restores hippocampal functions in mouse models of Alzheimer’s disease and has therapeutic implications for neurocognitive disorders. Proc Natl Acad Sci.

[224] Katsouri L (2015). Systemic administration of fibroblast growth factor-2 (FGF2) reduces BACE1 expression and amyloid pathology in APP23 mice. Neurobiol Aging.

[225] Stevens HE, Jiang GY, Schwartz ML, Vaccarino FM (2012). Learning and memory depend on fibroblast growth factor receptor 2 functioning in hippocampus. Biol Psychiatry.

[226] Garcia KO (2014). Therapeutic effects of the transplantation of VEGF overexpressing bone marrow mesenchymal stem cells in the hippocampus of murine model of Alzheimerâ€^TM^s disease. Front Aging Neurosci..

[227] Rafii MS (2018). Adeno-associated viral vector (Serotype 2)–nerve growth factor for patients with Alzheimer disease. JAMA Neurol.

[228] Hensley K (2010). Neuroinflammation in Alzheimer’s disease: mechanisms, pathologic consequences, and potential for therapeutic manipulation. J Alzheimer’s Dis.

[229] Gratuze M, Leyns CEG, Holtzman DM (2018). New insights into the role of TREM2 in Alzheimer’s disease. Mol Neurodegener.

[230] Hendrickx DAE, van Eden CG, Schuurman KG, Hamann J, Huitinga I (2017). Staining of HLA-DR, Iba1 and CD68 in human microglia reveals partially overlapping expression depending on cellular morphology and pathology. J Neuroimmunol.

[231] Hopperton KE, Mohammad D, Trépanier MO, Giuliano V, Bazinet RP (2018). Markers of microglia in post-mortem brain samples from patients with Alzheimer’s disease: a systematic review. Mol Psychiatry.

[232] Felsky D (2019). Neuropathological correlates and genetic architecture of microglial activation in elderly human brain. Nat Commun.

[233] Carter SF (2012). Evidence for astrocytosis in prodromal Alzheimer disease provided by 11C-deuterium-L-deprenyl: a multitracer PET paradigm combining 11C-Pittsburgh compound B and 18F-FDG. J Nucl Med.

[234] Kamphuis W (2012). GFAP isoforms in adult mouse brain with a focus on neurogenic astrocytes and reactive astrogliosis in mouse models of Alzheimer disease. Plos One.

[235] Verkhratsky A, Olabarria M, Noristani HN, Yeh C-Y, Rodriguez JJ (2010). Astrocytes in Alzheimer’s disease. Neurotherapeutics.

[236] Kashon ML (2004). Associations of cortical astrogliosis with cognitive performance and dementia status. J Alzheimer’s Dis.

[237] Osborn LM, Kamphuis W, Wadman WJ, Hol EM (2016). Astrogliosis: an integral player in the pathogenesis of Alzheimer’s disease. Prog Neurobiol.

[238] Lue L (2015). TREM 2 protein expression changes correlate with A lzheimer’s disease neurodegenerative pathologies in post-mortem temporal cortices. Brain Pathol.

[239] Lue L-F, Brachova L, Civin WH, Rogers J (1996). Inflammation, Aβ deposition, and neurofibrillary tangle formation as correlates of Alzheimer’s disease neurodegeneration. J Neuropathol Exp Neurol.

[240] Parachikova A (2007). Inflammatory changes parallel the early stages of Alzheimer disease. Neurobiol Aging.

[241] Vehmas AK, Kawas CH, Stewart WF, Troncoso JC (2003). Immune reactive cells in senile plaques and cognitive decline in Alzheimer’s disease. Neurobiol Aging.

[242] Ricciarelli R (2004). CD36 overexpression in human brain correlates with β-amyloid deposition but not with Alzheimer’s disease. Free Radic Biol Med.

[243] Haure-Mirande J-V (2017). Deficiency of TYROBP, an adapter protein for TREM2 and CR3 receptors, is neuroprotective in a mouse model of early Alzheimer’s pathology. Acta Neuropathol.

[244] Haure-Mirande J-V (2019). Integrative approach to sporadic Alzheimer’s disease: deficiency of TYROBP in cerebral Aβ amyloidosis mouse normalizes clinical phenotype and complement subnetwork molecular pathology without reducing Aβ burden. Mol Psychiatry.

[245] Moonen S (2023). Pyroptosis in Alzheimer’s disease: cell type-specific activation in microglia, astrocytes and neurons. Acta Neuropathol.

[246] Chen W-T (2020). Spatial transcriptomics and in situ sequencing to study Alzheimer’s disease. Cell.

[247] Wang W-Y, Tan M-S, Yu J-T, Tan L (2015). Role of pro-inflammatory cytokines released from microglia in Alzheimer’s disease. Ann Transl Med.

[248] Salter MW, Beggs S (2014). Sublime microglia: expanding roles for the guardians of the CNS. Cell.

[249] Sierra A (2010). Microglia shape adult hippocampal neurogenesis through apoptosis-coupled phagocytosis. Cell Stem Cell.

[250] Madore C, Yin Z, Leibowitz J, Butovsky O (2020). Microglia, lifestyle stress, and neurodegeneration. Immunity.

[251] Nakano M (2020). An enriched environment prevents cognitive impairment in an Alzheimer’s disease model by enhancing the secretion of exosomal microRNA-146a from the choroid plexus. Brain Behav Immun - Heal.

[252] Escartin C (2021). Reactive astrocyte nomenclature, definitions, and future directions. Nat Neurosci.

[253] Paolicelli RC (2022). Microglia states and nomenclature: a field at its crossroads. Neuron.

[254] Swerdlow RH (2018). Mitochondria and mitochondrial cascades in Alzheimer’s disease. J Alzheimer’s Dis.

[255] Kapogiannis D, Mattson MP (2011). Disrupted energy metabolism and neuronal circuit dysfunction in cognitive impairment and Alzheimer’s disease. Lancet Neurol.

[256] Sorrentino V (2017). Enhancing mitochondrial proteostasis reduces amyloid-β proteotoxicity. Nature.

[257] Adav SS, Park JE, Sze SK (2019). Quantitative profiling brain proteomes revealed mitochondrial dysfunction in Alzheimer’s disease. Mol Brain.

[258] Ionescu-Tucker A, Cotman CW (2021). Emerging roles of oxidative stress in brain aging and Alzheimer’s disease. Neurobiol Aging.

[259] Phongpreecha T (2021). Single-synapse analyses of Alzheimer’s disease implicate pathologic tau, DJ1, CD47, and ApoE. Sci. Adv..

[260] Schmidt MF, Gan ZY, Komander D, Dewson G (2021). Ubiquitin signalling in neurodegeneration: mechanisms and therapeutic opportunities. Cell Death Differ.

[261] Gatz M (2006). Role of genes and environments for explaining Alzheimer disease. Arch Gen Psychiatry.

[262] Lambert J-C (2013). Meta-analysis of 74,046 individuals identifies 11 new susceptibility loci for Alzheimer’s disease. Nat Genet.

[263] Jansen IE (2019). Genome-wide meta-analysis identifies new loci and functional pathways influencing Alzheimer’s disease risk. Nat Genet.

[264] Dubelaar EJG (2004). ApoE ɛ4 genotype is accompanied by lower metabolic activity in Nucleus Basalis of Meynert Neurons in Alzheimer patients and controls as indicated by the size of the Golgi apparatus. J Neuropathol Exp Neurol.

[265] Reiman EM (2020). Exceptionally low likelihood of Alzheimer’s dementia in APOE2 homozygotes from a 5,000-person neuropathological study. Nat Commun.

[266] Wang HX (2012). Education halves the risk of dementia due to apolipoprotein epsilon4 allele: a collaborative study from the Swedish brain power initiative. Neurobiol Aging.

[267] Kaup AR (2015). Cognitive resilience to apolipoprotein E ε4: contributing factors in black and white older adults. JAMA Neurol.

[268] Dekhtyar S (2019). Genetic risk of dementia mitigated by cognitive reserve: a cohort study. Ann Neurol.

[269] Wang H-X, MacDonald SWS, Dekhtyar S, Fratiglioni L (2017). Association of lifelong exposure to cognitive reserve-enhancing factors with dementia risk: a community-based cohort study. Plos Med.

[270] Sørensen M, Christensen K, Stevnsner T, Christiansen L (2009). The Mn-superoxide dismutase single nucleotide polymorphism rs4880 and the glutathione peroxidase 1 single nucleotide polymorphism rs1050450 are associated with aging and longevity in the oldest old. Mech Ageing Dev.

[271] Mengel-From J, Christensen K, McGue M, Christiansen L (2011). Genetic variations in the CLU and PICALM genes are associated with cognitive function in the oldest old. Neurobiol. Aging.

[272] Monsell SE (2017). Genetic comparison of symptomatic and asymptomatic persons with Alzheimer disease neuropathology. Alzheimer Dis Assoc Disord.

[273] Erickson CM (2019). KLOTHO heterozygosity attenuates APOE4-related amyloid burden in preclinical AD. Neurology.

[274] Belloy ME, Napolioni V, Han SS, Le Guen Y, Greicius MD (2020). Association of Klotho -VS heterozygosity with risk of Alzheimer disease in individuals who carry APOE4. JAMA Neurol.

[275] Ridge PG (2017). Linkage, whole genome sequence, and biological data implicate variants in RAB10 in Alzheimer’s disease resilience. Genome Med.

[276] Ward DD (2015). The BDNF Val66Met polymorphism moderates the relationship between cognitive reserve and executive function. Transl Psychiatry.

[277] Ward DD (2017). The *BDNF* Val66Met polymorphism moderates the effect of cognitive reserve on 36-month cognitive change in healthy older adults. Alzheimer’s Dement Transl Res Clin Interv.

[278] Kramer PL (2011). Alzheimer disease pathology in cognitively healthy elderly: a genome-wide study. Neurobiol Aging.

[279] van der Lee SJ (2019). A nonsynonymous mutation in PLCG2 reduces the risk of Alzheimer’s disease, dementia with Lewy bodies and frontotemporal dementia, and increases the likelihood of longevity. Acta Neuropathol.

[280] Sims R (2017). Rare coding variants in PLCG2, ABI3, and TREM2 implicate microglial-mediated innate immunity in Alzheimer’s disease. Nat Genet.

[281] White CC (2017). Identification of genes associated with dissociation of cognitive performance and neuropathological burden: Multistep analysis of genetic, epigenetic, and transcriptional data. Plos Med.

[282] Wetzel-Smith MK (2014). A rare mutation in UNC5C predisposes to late-onset Alzheimer’s disease and increases neuronal cell death. Nat Med.

[283] Lei H, Li J, Zhao Z, Liu L (2016). Inhibition of ectodermal-neural cortex 1 protects neural cells from apoptosis induced by hypoxia and hypoglycemia. J Mol Neurosci.

[284] Van Deerlin VM (2010). Common variants at 7p21 are associated with frontotemporal lobar degeneration with TDP-43 inclusions. Nat Genet.

[285] Reed BR (2010). Measuring cognitive reserve based on the decomposition of episodic memory variance. Brain.

[286] Mukherjee S (2012). Genetic architecture of resilience of executive functioning. Brain Imaging Behav.

[287] Mukherjee S (2014). Gene-based GWAS and biological pathway analysis of the resilience of executive functioning. Brain Imaging Behav.

[288] Nho K (2019). Altered bile acid profile in mild cognitive impairment and Alzheimer’s disease: relationship to neuroimaging and CSF biomarkers. Alzheimer’s Dement.

[289] Wood PL (2015). Targeted lipidomics of fontal cortex and plasma Diacylglycerols (DAG) in mild cognitive impairment and Alzheimer’s disease: validation of DAG accumulation early in the pathophysiology of Alzheimer’s disease. J Alzheimer’s Dis.

[290] Hebert LE, Weuve J, Scherr PA, Evans DA (2013). Alzheimer disease in the United States (2010–2050) estimated using the 2010 census. Neurology.

[291] Nebel RA (2018). Understanding the impact of sex and gender in Alzheimer’s disease: a call to action. Alzheimer’s Dement.

[292] Guo L, Zhong MB, Zhang L, Zhang B, Cai D (2022). Sex Differences in Alzheimer’s disease: insights from the multiomics landscape. Biol Psychiatry.

[293] Granger MW (2016). A TgCRND8 mouse model of Alzheimer’s disease exhibits sexual Dimorphisms in behavioral indices of cognitive reserve. J Alzheimer’s Dis.

[294] Johnson NR (2023). CSF1R inhibitors induce a sex-specific resilient microglial phenotype and functional rescue in a tauopathy mouse model. Nat Commun.

[295] Hu Y (2021). Sex differences in the neuropathological hallmarks of Alzheimer’s disease: focus on cognitively intact elderly individuals. Neuropathol Appl Neurobiol.

[296] Chen X (2023). Sexually dimorphic age-related molecular differences in the entorhinal cortex of cognitively intact elderly: relation to early Alzheimer’s changes. Alzheimer’s Dement.

[297] Tijms BM (2024). Cerebrospinal fluid proteomics in patients with Alzheimer’s disease reveals five molecular subtypes with distinct genetic risk profiles. Nat Aging.

[298] Musi N (2018). Tau protein aggregation is associated with cellular senescence in the brain. Aging Cell.

[299] Gonzales, M. M. et al. Senolytic Therapy to Modulate the Progression of Alzheimer’s Disease (SToMP-AD): A Pilot Clinical Trial. J Prev Alzheimer’s Dis 1–8 (2021). 10.14283/jpad.2021.62. 10.14283/jpad.2021.62 PMC8612719 35098970

